# Effect of spatio-temporal shifts in salinity combined with other environmental variables on the ecological processes provided by *Zostera noltei* meadows

**DOI:** 10.1038/s41598-017-01359-2

**Published:** 2017-05-02

**Authors:** Ana I. Sousa, Ricardo Calado, Daniel F. R. Cleary, Cláudia Nunes, Manuel A. Coimbra, João Serôdio, Ana I. Lillebø

**Affiliations:** 1 Department of Biology & CESAM – Centre for Environmental and Marine Studies, University of Aveiro, Campus Universitário de Santiago, 3810-193 Aveiro, Portugal; 2 CICECO, University of Aveiro, Campus Universitário de Santiago, 3810-193 Aveiro, Portugal; 3 QOPNA, University of Aveiro, Campus Universitário de Santiago, 3810-193 Aveiro, Portugal

## Abstract

The present study aims to assess the plastic response of *Zostera noltei* meadows traits under spatio-temporal shifts in salinity combined with sediment environmental variables (temperature; pH; loss-on-ignition (LOI); carbon (C) and nitrogen (N) pools (top 5 cm)). *Z*. *noltei* biomass, C and N pools, leaf photosynthetic performance and esterified fatty acid (FA) profile were assessed within a temperate coastal lagoon during winter and late spring, along sites spatially distributed. None of the surveyed traits for *Z*. *noltei* displayed a clear spatial trend. *Z*. *noltei* proved to be euryhaline, whose biology was only slightly affected within this salinity range, in each season (14–39 in winter; 33–41 in late spring). Seasonal differences in salinity and environmental parameters explain the differences recorded in *Z*. *noltei* traits (aboveground biomass, N and C pools; photosynthetic performance). Spatio-temporal salinity shifts did not significantly affect the pool of FA present in *Z*. *noltei*. Overall, within the salinity range surveyed, the ecological processes studied and regulating *Z*. *noltei* meadows do not appear to be at risk. This work reinforces the plasticity of *Z*. *noltei* to salinity shifts within the studied range, with this finding being particularly relevant in the context of extreme weather events (e.g., winter freshwater floods, summer droughts).

## Introduction

Seagrass meadows have a worldwide distribution from tropical to temperate environments^1^. As primary producers, their productivity is extremely high when compared to other ecosystems^2, 3^. Seagrass meadows play a key role in significant ecosystem functions and ecological processes^2^, and provide important ecosystem services (ES). *Zostera noltei* Hornemann (1832) meadows contribute to sediment cohesiveness and reduce tidal and storm erosion and sediment resuspension (stabilizing effect)^4, 5^, having an important role in nutrient cycling as well^6^. Overall, these habitats contribute to human well-being, from local to global level by providing nursery areas for commercially valuable seafood species and helping to regulate the global carbon (C) cycle through C sequestration^7–9^.

Despite providing key ecological processes and services, seagrass meadows are globally declining, mostly due to multiple, direct and indirect, human-related perturbations^10^. In fact, some seagrass species are even at risk of extinction^11^. Eutrophication, as a result of anthropogenic activity, has been pointed out as one of the main drivers of seagrass decline worldwide^10, 12^. Other important drivers that have contributed to decline include physical disturbance^13, 14^ and elevated temperature^15^. Multiple-interacting factors such as changes in ammonium concentration, light reduction in the water column due to dredging activities, grazing pressure and herbicides, may also contribute to seagrass decline^16^.

*Z*. *noltei* is geographically widespread along the eastern Atlantic coastline (from Sweden to Mauritania)^7, 17^. It is a perennial seagrass species and mostly intertidal, but can also occur in subtidal habitats. It is able to tolerate a wide salinity range^18^ and is commonly classified as euryhaline^19^. *Z*. *noltei* has the ability to morphologically and physiologically acclimate to different environmental conditions including fluctuations in physical (e.g., wave exposure, sediment resuspension, turbidity, and sediment particle size) and chemical parameters (salinity changes, nutrient and light availability)^20–22^.

Despite these features, shifts in salinity have been shown to impact and structure seagrass communities. Hypo- or hypersaline conditions can affect several seagrass species by triggering shifts in physiological processes, such as photosynthesis and respiration, osmoregulatory processes, carbon metabolism, and nutrient uptake (see ref. 23 for a review; ref. 24). Moreover, fatty acid (FA) synthesis in seagrasses might be affected by salinity shifts, as recorded for seaweeds^25^. Other environmental parameters such as temperature^26, 27^ and light^28^, are also known to influence FA synthesis. FA signatures reflect physiological changes in seagrasses and may reveal stress conditions due to diverse environmental settings. Namely, linoleic (18:2*n*-6) and alpha-linolenic acid (18:3*n*-3), recognized as seagrass (and vascular plants) biomarkers^29, 30^, can reflect environmental variations by changing their unsaturation level and relative content.

Therefore, shifts in salinity may affect seagrass biology and physiology. In turn, this will impact the ecological processes and functions supporting the ES provided by these meadows. Particularly, primary production (through photosynthesis), nutrient cycling (through nitrogen (N) and phosphorous (P) uptake) and C cycle regulation (through C sequestration and storage), as well as FA synthesis, are some of the physiological processes and functions likely to be affected by salinity shifts. Other studies have shown that seedling germination of *Z*. *noltei* increases at low salinity values^31^ and that shoots are more tolerant to lower salinities than to hypersalinity^22^. Seawater salinity (35) has also been shown to trigger mortality in *Z*. *noltei* populations^18^. With the expected increase in the frequency of extreme weather events promoted by global climate change^32, 33^, salinity in winter can drop to zero due to heavy freshwater floods, while dry hot summers may drive salinity to reach values exceeding those of seawater (e.g. in mesotidal system during low tide).

The main objective of the present study was to assess to what extent spatio-temporal variation in salinity, combined with sediment temperature, pH, LOI (loss-on-ignition) and the sediment pool of C and N on the top 5 cm, affect ecological processes supporting ES provided by *Z*. *noltei* meadows. To address this objective authors formulated the following null (H_0_) hypotheses: H_0_1 - high salinity does not affect photosynthetic performance, C metabolism and nutrient uptake; H_0_2 - ecological functions and ES provided by *Z*. *noltei* meadows, namely biomass provisioning (primary production), nutrient cycling (e.g. storage) and climate regulation (through C fixation and storage) are not affected by salinity shifts and other environmental variables; and H_0_3 - FA unsaturation level is not affected under salt stress and temperature decrease.

To address this topic we measured the spatio-temporal shifts in: above- and belowground biomass, N and C pools, photosynthetic performance and FA composition in *Z*. *noltei*. Sampling was performed following the *Z*. *noltei* distribution along the Mira channel, part of a shallow coastal lagoon (Ria de Aveiro, Portugal). *Z*. *noltei* traits were used as a proxy of the status of ecological processes supporting regulation and maintenance ES provided by these meadows. Given the wide geographical distribution of this seagrass species, these results will be applicable to other systems with similar environmental ranges and consequently contribute to a better understanding of *Z*. *noltei* meadows’ dynamics.

## Materials and Methods

### Study site

**Figure 1:**
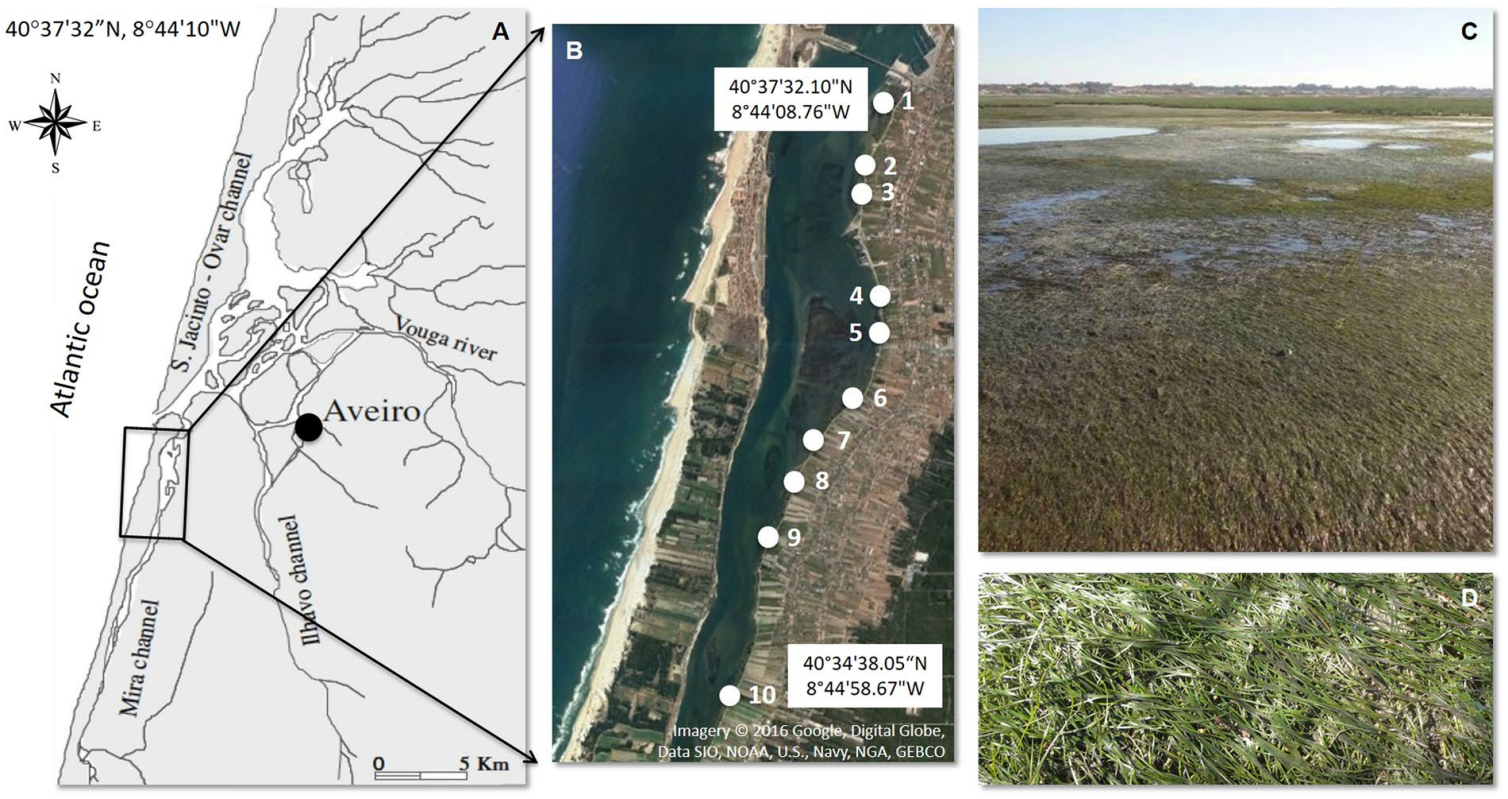
Mira channel at Ria de Aveiro coastal lagoon (Portugal) (**A**,**B**) sampling sites location (from site 1 (40°37′32.10″N, 8°44′08.76″W) to site 10 (40°34′38.05″N, 8°44′58.67″W)). (**C**) – *Z*. *noltei* meadow overview at the sampling site 5; (**D**) – *Z*. *noltei* detail. Map generated with ArcGIS 10, (http://www.esri.com/software/arcgis). Credits of World Terrain Base: Esri, USGS, NOAA, DeLorme, NPS. Mira channel side scheme adapted from: Imagery © 2016 Google, Digital Globe, Data SIO, NOAA, U.S., Navy, NGA, GEBCO. Pictures copyright: ©A.I. Lillebø and A.I. Sousa.

Ria de Aveiro, a LTER site (Long Term Ecosystem Research; http://www.lter-europe.net/), is a temperate shallow coastal lagoon located on the western coast of Portugal (40°38°N, 8°44°W). It has a complex geometry, forming four main channels with several branches, islands, inner basins and mudflats. In the south, the lagoon forms an elongated channel (about 20 km long), the Mira channel, which is described as having the characteristics of an estuary itself^34^. This shallow channel is characterized by a salinity gradient during high tide^35, 36^; where salinity stratification is not common^34^, unless there is a high freshwater input either from water runoff or heavy rainfall. However, it can be described as a poikilohaline water body^37^, since water salinity shows considerable spatio-temporal shifts of tidal and seasonal origin^34^. Sampling sites were located along the Mira channel between the two-homoiohaline environments^37^, i.e., from downstream, close to the source of marine water; to upstream towards the freshwater source, the Mira river, following the seagrass distribution (Fig. 1).

### Sampling strategy

*Z*. *noltei* was sampled along 5.5 km of the Mira channel, following the seagrass distribution (Fig. 1), in winter (February) and late spring (June) 2013. A total of 80 seagrass samples were collected across ten sites in spring tides during low tide, using Ø8 cm corers (50 mm depth) (four replicates at each site); samples were subsequently taken to the laboratory. Sediment pH and temperature were measured *in situ* at each sampling site and date, using a WTW–pH 330i/set equipped with SenTix® 41 (WTW, Weilheim, Germany). Water salinity was also measured *in situ* at each site along the channel in low water pools using a WTW Conductivity meter 330i/set equipped with Tetracon^®^ 325 probe (WTW, Weilheim, Germany), using the Practical Salinity Scale.

At the laboratory, *Z*. *noltei* roots and rhizomes were separated from rhizosediment. Aboveground (shoots/leaves) and belowground (roots and rhizomes) parts of the seagrass were separated, rinsed with distilled water and weighed. A small portion was freeze-dried and stored at −80 °C for FA analysis and the remainder was dried at 60 °C until constant weight. Rhizosediment was air dried, ground, and passed through a 0.25 mm mesh. Plant material was also ground and homogenised for subsequent analyses. Rhizosediment was characterized for particle size, interstitial water salinity, dry bulk density, organic matter (OM) content through loss-on-ignition (LOI%), and total C and N content. Sediment particle size (granulometry) was assessed once, as seasonal variations are not expected, by sequential sieving of the 0–50 mm depth sediment cores and classified according to Blott and Pye^38^. Interstitial water was extracted from the sediment using centrifugation and salinity was measured using a refractometer. Dry bulk density was assessed with the equation:

1 $${\rm{d}}({\rm{g}}.{{\rm{cm}}}^{-3})={{\rm{W}}}_{{\rm{d}}}/{{\rm{V}}}_{{\rm{t}}}$$

W_d_ being the dry weight of the sample (g) and V_t_ the total volume of the sample (cm^3^) (Vt = volume of the particles + volume of the water). Sediment LOI, as a proxy for OM was quantified after 8 h combustion at 500 °C, in prior dried sediment (105 °C, until constant weight).

Total carbon (C) and nitrogen (N) content in rhizosediment and *Z*. *noltei* (aboveground and belowground biomass) were quantified in a CHNS/O analyser (Fisons Instruments Model EA 1108, Beverly, Massachusetts, USA). *Z*. *noltei* N and C pools (mg m^−2^) were estimated by multiplying biomass (mg DW m^−2^) per N or C content (mg mg^−1^ DW).

### Photosynthetic performance

Once in the laboratory, *Z*. *noltei* samples were immersed in water, collected from each sampling site and maintained overnight at 18–20 °C under a dark:light cycle (12:12 h, L:D). The following day, photosynthetic performance of *Z*. *noltei* leaves was assessed by rapid light-response curves (RLCs) of photosystem II (PSII) relative electron transport rate (rETR) using a PAM fluorometer. This included a computer-operated PAM-Control Unit (Walz, Effeltrich, Germany) and a WATER-EDF-Universal emitter-detector unit (Gademann Instruments GmbH, Wurzburg, Germany). The fluorometer light source (measuring, actinic and saturating light) is a modulated blue light (LED-lamp peaking at 450 nm, half-bandwidth of 20 nm)^39^. The light delivered by the fluorometer and the fluorescence emitted by the leaf were conducted by a Ø1.5 mm plastic optical fibre. The optical fibre was maintained at a constant distance (1 mm) from the leaf. For each site, five *Z*. *noltei* shoots were selected and light adapted (70 μmol photons m^−2^ s^−1^) for a minimum of 15 min. RLCs were generated by subjecting the second youngest (innermost) leaf of each shoot (5 cm above the sheath (meristem) to 44 μmol photons m^−2^ s^−1^ for 2 min, followed by a sequence of increasing actinic light intensities (100, 134, 181, 261, 371, 515, 705 and 1149 μmol photons m^−2^ s^−1^). Each actinic light intensity was maintained for 10 s, after which a saturating light pulse was applied and fluorescence levels F_s_ and ${{\rm{F}}}_{{\rm{m}}}^{^{\prime} }$ were quantified. The effective quantum yield of PSII^40^ was calculated from:

2 $${{\rm{\Delta }}{\rm{F}}/{\rm{F}}}_{{\rm{m}}}^{^{\prime} }=({{\rm{F}}}_{{\rm{m}}}^{^{\prime} }-{{\rm{F}}}_{{\rm{s}}}){/{\rm{F}}}_{{\rm{m}}}^{^{\prime} },$$

and rETR was calculated as

3 $${\rm{rETR}}={{\rm{\Delta }}{\rm{F}}/{\rm{F}}}_{{\rm{m}}}^{^{\prime} }\times {\rm{E}}\times 0.5$$

E is the incident photosynthetic active irradiance and 0.5 is the fraction of photons absorbed by PSII (assuming that both photosystems absorb equal amount of photons; ref. 41). The model of Eilers and Peeters^42^ was fitted to rETR *vs* E curves, estimating the maximum photosynthetic efficiency (α; the initial slope of the curve), maximum relative electron transport rate (rETR_m_), photoacclimation index (E_k_) and optimum irradiance (E_opt_).

### Esterified fatty acid analyses

Esterified fatty acids (FA) were extracted from *Z*. *noltei* above- and belowground biomass, from sites 1, 2, 5, 7 and 10. The chosen sampling sites are geographically widespread along the sampling area and include the most upstream and the most downstream sites.

FA were extracted and converted into fatty acid methyl esters (FAMEs) through transesterification, following the method described by Aued-Pimentel *et al*.^43^. Freeze-dried *Z*. *noltei* samples (100 mg) were dispersed in 1 mL of *n*-hexane, to extract the FA, containing 0.375 g L^−1^ of heneicosanoic acid methyl ester (Sigma-Aldrich, Steinheim, Germany), as internal standard. A methanolic solution of 2 M KOH (0.2 mL) and saturated NaCl solution (2 mL) were added to the mixture. After intense vortexing and centrifugation for 5 min (2000 rpm), the supernatant was collected and dried in centrifugal evaporator (SpeedVac). Then, FAMEs were dissolved in *n*-hexane and 2 μL of this solution were analysed by gas chromatography (GC) equipped with flame ionization detector (FID) on a Perkin Elmer Clarus 400 equipment. The GC oven was programmed from an initial temperature of 50 °C, standing at this temperature for 3 min and following a linear increase to 180 °C at 25 °C min^−1^, a linear increase at 40 °C min^−1^ to 260 °C and maintain this temperature for 3 min. Hydrogen was the carrier gas at a flow rate of 1.7 mL min^−1^. The column used was DB1 with 30 m length, internal diameter of 0.25 mm and 0.10 μm film thickness (J&W Scientific, Folsom, CA). Data acquisition and analysis were carried out with TotalChrom Navigator Software. Supelco® 37 component FAME mix (Sigma-Aldrich, Steinheim, Germany) was used as standard for the identification by retention time and quantification of FAMEs by obtaining calibration curves in relation to the internal standard.

Some *Z*. *noltei* samples were run through a gas chromatography column coupled with a mass spectrometer (GC-MS) analyser for confirmation of FAMEs identification. An Agilent Technologies 6890 N Network (Santa Clara. CA) equipped with a DB-1 column (30 m length, 0.25 mm internal diameter and 0.1 μm film thickness) was used. The samples were injected in the injection port at 250 °C lined with a 4.0 mm i.d. splitless glass liner. The detector starts to operate after 5 min of injection (solvent delay). The GC-MS was connected to an Agilent 5973 Network Mass Selective Detector operating with an electron impact mode at 70 eV and scanning the range *m*/*z* 40–500 in a 1 s cycle in a full scan mode acquisition. The oven temperature programme was the same used in the GC-FID equipment. Helium was used as carrier gas with a column head pressure of 12 psi. The injector ion source and the transfer line were kept at 230 °C. Each FAME peak was integrated using the equipment’s software, and identified considering the retention time and mass spectrum of each FAME when compared to the Supelco®37 component FAME mix (Sigma-Aldrich, Steinheim, Germany). The FAMEs identification was confirmed by comparison with the chemical database Wiley and the spectral library “The AOCS Lipid Library”.

The concentration of each FA was calculated based on the concentration of the internal standard (heneicosanoic acid methyl ester). Values are expressed as net FA concentration (μg g^−1^ or mg g^−1^) and as relative percentages of the total pool of FA. FA profile is shown as individual FA, and as three FA classes: SFA (saturated fatty acids), MUFA (monounsaturated fatty acids) and PUFA (polyunsaturated fatty acids - FA with ≥ 2 double bonds).

### Statistical analyses

Two square matrices were imported into R (R Core Team 2013) using the read.table() function and containing data on *Z*. *noltei* traits (above- and belowground biomass; above- and belowground C pool, above- and belowground N pool; and photosynthetic parameters) and measured environmental variables (salinity (interstitial water), sediment LOI, pH, temperature; sediment C pool; sediment N pool). The square matrix of species traits was first log_e_ (*x* + 1) transformed (in order to normalize the distribution of the data) and three distance matrices constructed using the Euclidean index with the vegdist() function in the vegan package^44^ in R. The distance matrices contained 1. all data, 2. data from February only and 3. data from June only. After controlling for normality with a Shapiro test and homogeneity of variance with a Bartlett test in R (most variables deviated significantly for both measures), we tested for significant differences in *Z*. *noltei* aboveground biomass (AbB), aboveground C pool (AbC), aboveground N pool (AbN), belowground biomass (BeB), belowground C pool (BeC), belowground N pool (BeN), maximum photosynthetic efficiency (α = Pal), maximum relative electron transport rate (rETR_m_ = Pmx), photoacclimation index (E_k_ = Pek), optimum irradiance (E_opt_ = Pop), sediment C pool (SdC) and sediment N pool (SdN) between seasons using a Friedman test with the friedman.test function in R. We subsequently assessed the relationship between temperature, pH and salinity with AbB, AbC, AbN, BeB, BeC, BeN, Pal, Pmx, Pek, Pop. We used the lm function in R to test for linear relationships between these variables and temperature, pH and salinity with separate tests performed for each sampling event (winter (February) and late spring (June)). Variation among sample sites was assessed with Principal Coordinates Analysis (PCO) using the cmdscale() function in R with the Euclidean distance matrices as input. Weighted averages scores were computed for *Z*. *noltei* traits on the first two PCO axes using the wascores() function in the vegan package. Measured variables, namely sediment temperature, LOI, pH, sediment pools of C and N at the top 5 cm, and salinity were fit onto the PCO ordinations using the envfit() function in vegan. Using the envfit() function, we also tested for significant relationships between these variables and the PCO ordinations of *Z*. *noltei* traits using 999 permutations; all other arguments in the function were left as default. Detailed descriptions of the functions used here can be found in R (e.g., ?cmdscale) and online in the reference manuals (e.g., http://cran.r-project.org/web/packages/vegan/index.html; checked 2014 09 21). A *t*-test was performed for differences on C:N ratio between seasons (winter vs late spring) in aboveground and belowground *Z*. *noltei*, using the Statistics software package.

*Z*. *noltei* esterified fatty acid (FA) variation among different sites and seasons was tested through PERMDISP (tests the homogeneity of multivariate dispersions), which was performed after a log (x + 1) transformation to the FA net concentrations and after building a resemblance matrix considering Bray-Curtis similarity coefficients. PERMDISP was run on the basis of distances to centroids. A SIMPER (Similarity Percentage) analysis was also run once differences were obtained among sites, in order to identify which FA mostly explained dissimilarities among sites in *Z*. *noltei* FA profiles. All FA analyses were performed using PRIMER v.6.0 and PERMANOVA + add on (PRIMER-E Ltd., Plymouth, UK).

## Results

### Sediment characterization

During winter, a typical estuarine salinity gradient was observed in the sediment interstitial water along the Mira channel. However, this gradient was disturbed by spatial shifts, due to freshwater input at some sites (Table 1). In late spring, the salinity range was much lower than that recorded in the winter (ranging from 33.0 ± 1.4 to 40.5 ± 1.0 and 14.0 ± 1.0 to 39.0 ± 2.0, respectively). Reflecting the freshwater influence pH in the rhizosediment was lower in late spring than winter (Table 1). Sediment particle size (granulometry) ranged from sandy-mud [clay + silt (<63 μm) >50%; sand (>63 μm) <50%] to muddy-sand [sand >50%; clay + silt <50%] and did not show a clear spatial trend. Sites 2, 3 and 10 showed coarser sediments and sites 4 and 5 had a finer granulometry (Table 1). Total C and N rhizosediment pools in the top 5 cm were higher in winter when compared to late spring (Table 1).

**Table 1:** Physicochemical characterization of *Zostera noltei* rhizosediment (0–50 mm depth for interstitial water salinity, pH, LOI, granulometry, C and N pool; mean ± SD), for each sampling site, in winter (W) and late spring (S).

Site	Sampling season	Rhizosediment						
		Salinity	pH	Tempera-ture (°C)	LOI (%)	Particle size ^(**1**)^	C pool (g m^−2^ cm^−1^)	N pool (g m^−2^ cm^−1^)
1	W	39.0 ± 2.0	7.1 ± 0.1	15.6	3.8 ± 0.2	—	185 ± 15	10.2 ± 0.8
	S	40.3 ± 0.5	6.9 ± 0.1	18.4 ± 0.6	4.3 ± 00.4	mS (55% S; 45% St&C)	23 ± 2	2.9 ± 0.2
2	W	32.3 ± 2.6	7.3 ± 0.0	13.8	2.6 ± 0.1	—	176 ± 16	21.3 ± 1.9
	S	33.0 ± 1.4	7.0 ± 0.1	18.6 ± 1.0	2.3 ± 0.3	(m)S (81% S; 19% St&C)	14 ± 1	3.5 ± 0.4
3	W	32.3 ± 2.6	8.2 ± 0.0	14.1	1.7 ± 0.0	—	110 ± 10	2.6 ± 0.2
	S	39.8 ± 1.3	7.5 ± 0.4	23.3 ± 5.3	3.1 ± 0.2	mS (66% S; 34% St&C)	25 ± 2	6.0 ± 0.6
4	W	26.3 ± 1.3	8.1 ± 0.1	13.4	3.1 ± 0.1	—	177 ± 14	4.1 ± 0.3
	S	38.5 ± 0.6	7.1 ± 0.2	19.7 ± 1.6	3.6 ± 0.2	sM (38% S; 62% St&C)	17 ± 2	3.7 ± 0.3
5	W	14.0 ± 1.0	8.1 ± 0.0	12.7	1.6 ± 0.0	—	87 ± 7	9.7 ± 0.7
	S	38.3 ± 1.0	7.4 ± 0.1	22.6 ± 1.1	4.7 ± 0.2	sM (39% S; 61% St&C)	21 ± 2	1.4 ± 0.1
6	W	24.5 ± 0.6	8.1 ± 0.0	14.4	4.3 ± 0.1	—	186 ± 17	4.3 ± 0.4
	S	40.3 ± 0.5	6.9 ± 0.3	22.9 ± 1.0	3.9 ± 0.9	mS (57% S; 43% St&C)	24 ± 2	6.2 ± 0.6
7	W	28.3 ± 2.2	7.2 ± 0.1	12.2	2.6 ± 0.8	—	160 ± 11	19.6 ± 1.3
	S	40.3 ± 0.5	7.1 ± 0.1	18.4 ± 2.0	5.2 ± 0.3	sM (47% S; 53% St&C)	21 ± 2	1.5 ± 0.2
8	W	26.5 ± 1.3	7.6 ± 0.2	13.8	3.8 ± 0.2	—	264 ± 18	38.3 ± 2.6
	S	38.8 ± 1.0	7.0 ± 0.2	17.8 ± 0.4	4.5 ± 0.3	sM (48% S; 52% St&C)	20 ± 2	1.8 ± 0.2
9	W	23.5 ± 1.0	7.4 ± 0.3	15	3.9 ± 0.1	—	209 ± 18	35.2 ± 3.0
	S	40.5 ± 1.0	7.1 ± 0.1	17.7 ± 0.4	4.2 ± 0.0	sM (48% S; 52% St&C)	18 ± 2	4.1 ± 0.5
10	W	17.0 ± 2.0	7.0 ± 0.0	14.5	1.4 ± 0.2	—	76 ± 6	2.2 ± 0.2
	S	38.0 ± 1.2	7.1 ± 0.1	14.1 ± 0.5	3.6 ± 0.4	mS (60% S; 40% St&C)	20 ± 2	3.0 ± 0.3

^(1)^ According to Blott and Pye (2012)^38^ classification (mS: muddy sand (sand >50% and clay + silt <50%); (m)S: slightly muddy sand; sM: sandy mud (clay + silt >50% and sand <50%)). Relative percentages of sand (S: grain size >63 μm) and mud (silt&clay, St&C: grain size <63 μm) are shown within brackets for each study site.

### Seasonal changes in *Zostera noltei* traits and environmental parameters

**Figure 2:**
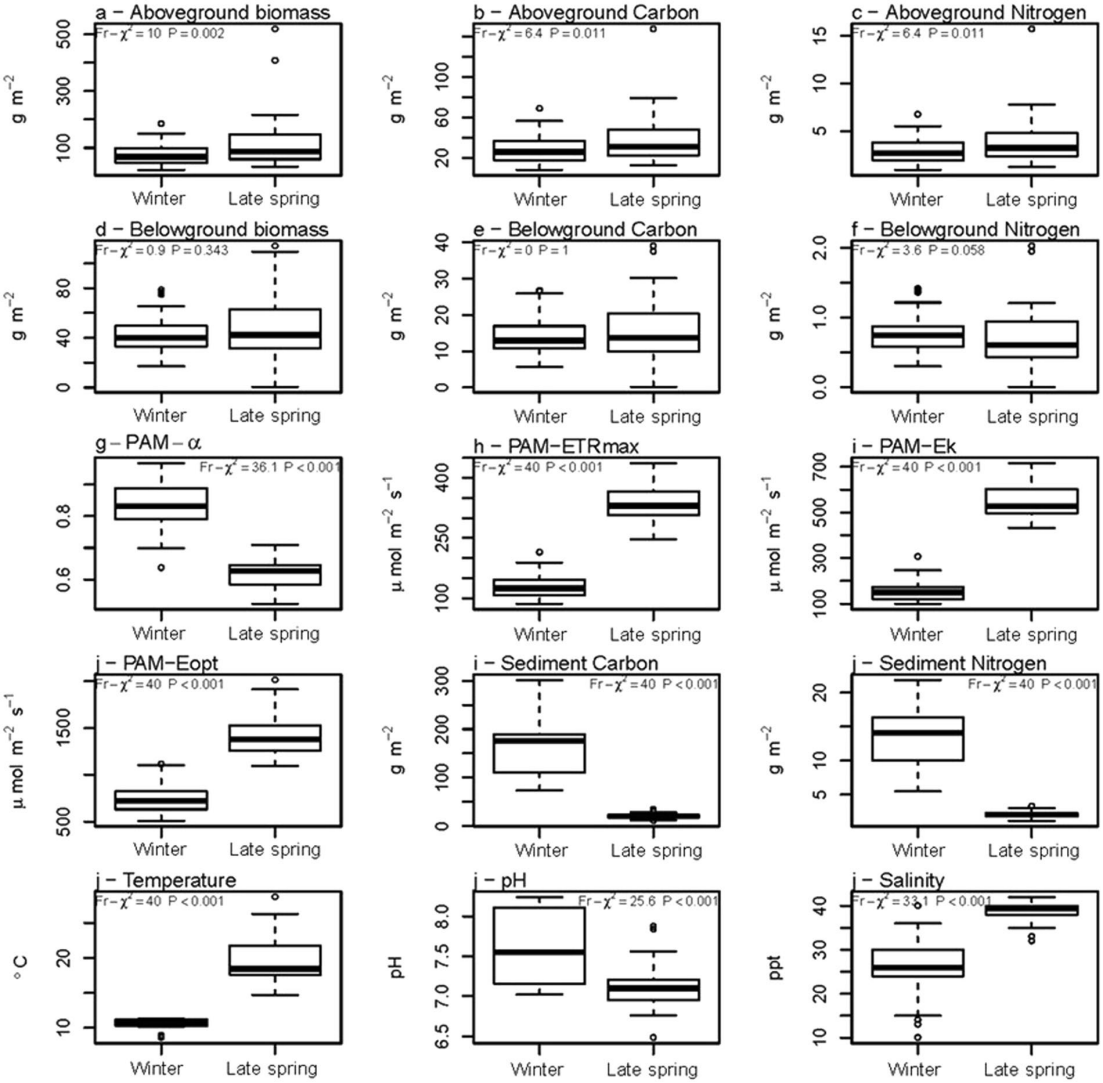
*Zostera noltei* traits and environmental variables in winter and late spring. Friedman test statistical results are also shown. (**a**): aboveground biomass (gDW m^−2^), (**b**): aboveground C pool (gC m^−2^), (**c**): aboveground N pool (gN m^−2^), (**d**): belowground biomass (gDW m^−2^), (**e**): belowground C pool (gC m^−2^), (**f**): belowground N pool (gN m^−2^); photosynthetic parameters – (**g**): maximum photosynthetic efficiency (α, μmol^−1^ m^2^ s^1^), (**h**): maximum relative electron transport rate (rETR), (**i**): photoacclimation index (E_k_, μmol m^−2^ s^−1^), (**j**): optimum irradiance (E_opt_, μmol m^−2^ s^−1^); environmental parameters – (**j**): sediment C pool (gC m^−2^ cm^−1^), (**k**): sediment N pool (gC m^−2^ cm^−1^), sediment temperature (°C), sediment pH, sediment (interstitial water salinity).

**Figure 3:**
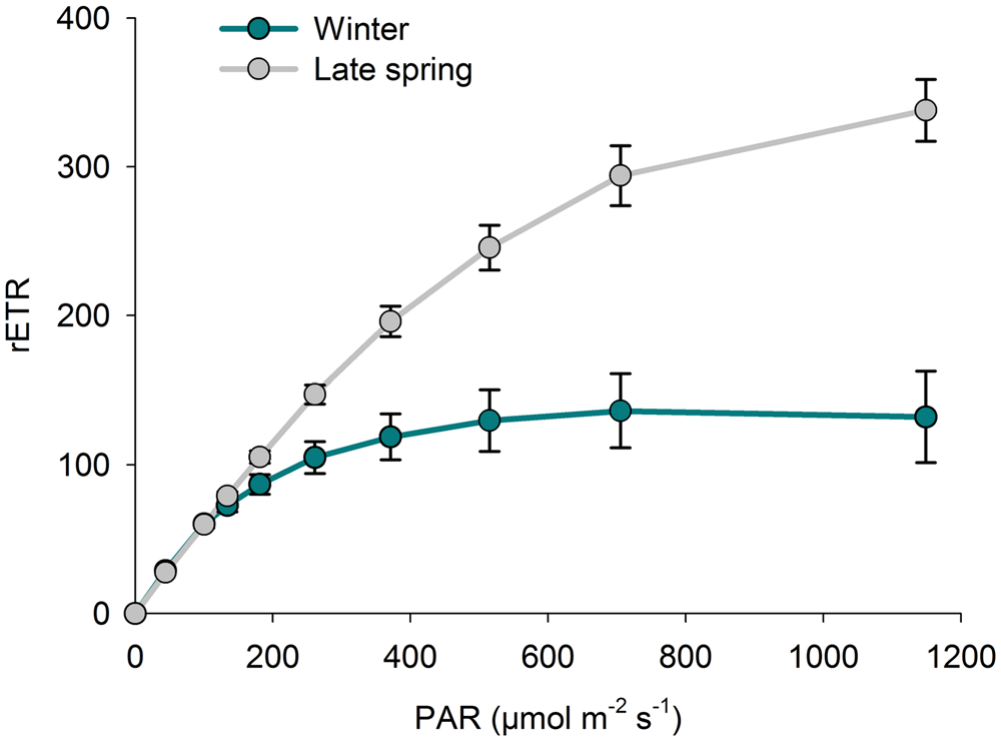
Representative light-response curves of relative electron transport rate of PSII (rETR) measured on *Z*. *noltei* leaves (collected in winter and late spring) at sampling site 5 (N = 5, mean ± SD).

There were significant differences (Friedman test, *p* < 0.05) between seasons in *Z*. *noltei* aboveground biomass (Fig. 2a), aboveground C pool (Fig. 2b), aboveground N pool (Fig. 2c), photosynthetic efficiency (α) (Fig. 2g), maximum rETR (rETRm) (Figs 2h and 3), Ek (Fig. 2i) and Eopt (Fig. 2j) and sediment C pool (Fig. 2k) and N pool (Fig. 2l). There were, however, no significant differences in *Z*. *noltei* belowground biomass (Fig. 2d), C pool (Fig. 2e) or N pool (Fig. 2f) between seasons (Friedman test, *p* > 0.05).

The relative contribution of belowground material to the total biomass in winter ranged from 31 to 45% (average 37%) and from 23% to 47% (average 33%) in late spring. Most sites displayed more than 70% of aboveground biomass in late spring. There were no differences on C:N ratio in the aboveground biomass in winter (9.4 ± 0.5) and late spring (9.9 ± 0.9) (*t*-test, *t* = −1.636, *p* > 0.05). The C:N ratio of the belowground biomass increased from winter (18.2 ± 1.0) to late spring (23.5 ± 4.1) (*t*-test, *t* = −3.936, *p* < 0.001). *Z*. *noltei* C and N pools were calculated considering both the biomass and C or N content. Thus, C and N pools reflected the seasonal and spatial pattern of these parameters.

**Figure 4:**
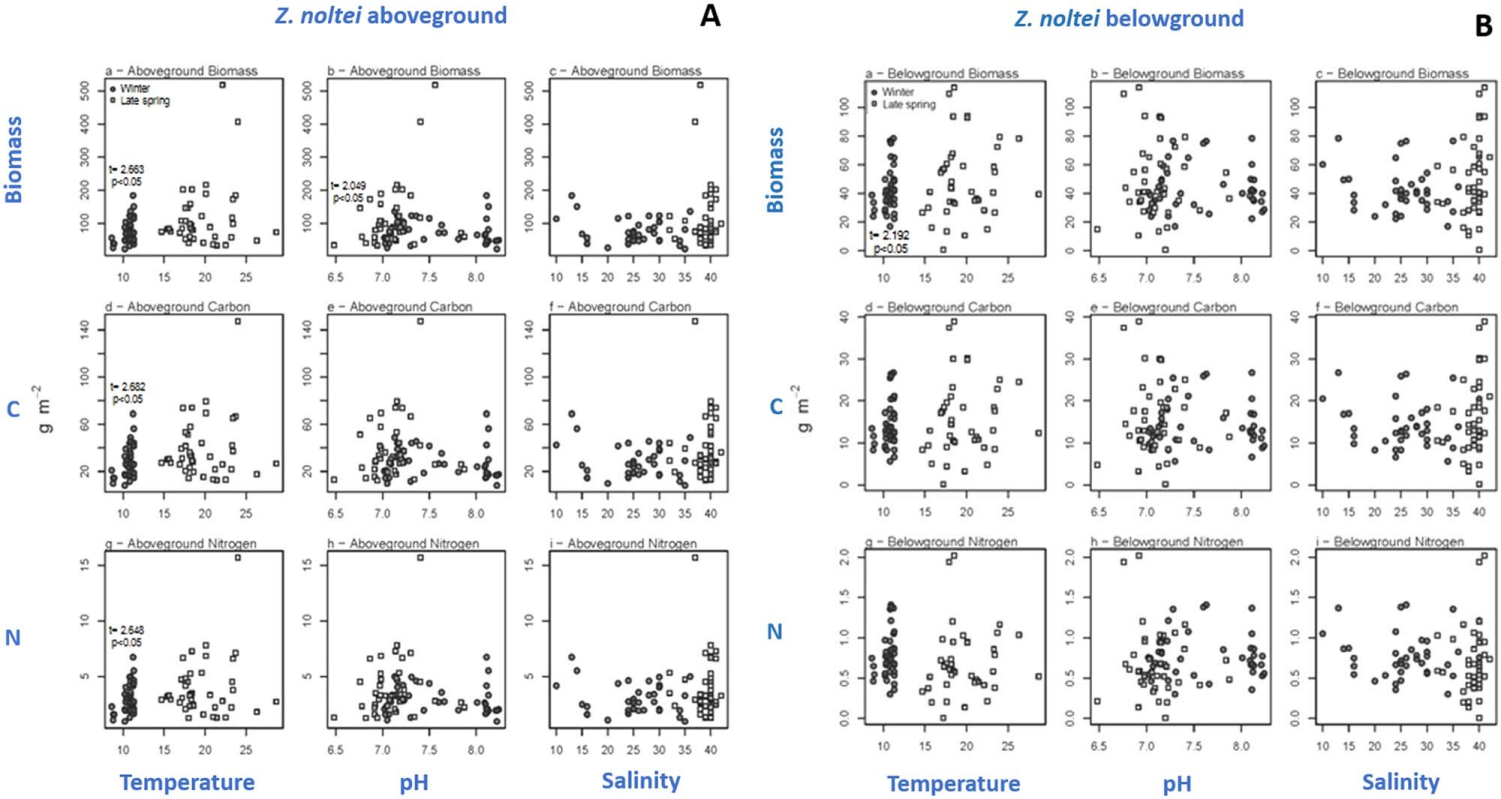
*Zostera noltei* biomass (gDW m^−2^) *versus* (**a**) temperature, (**b**) pH and (**c**) salinity in winter and late spring; C pool (gC m^−2^) *versus* (**d**) temperature, (**e**) pH and (**f**) salinity; and aboveground N pool (gN m^−2^) *versus* (**g**) temperature, (**h**) pH and (**i**) salinity. Statistical significant linear relationships within each season are shown (t value; p < 0.05). A - refers to *Z*. *noltei* aboveground biomass, C and N pools; B - refers to *Z*. *noltei* belowground biomass, C and N pools. All statistical results are shown as Supplementary information (Table S1).

**Figure 5:**
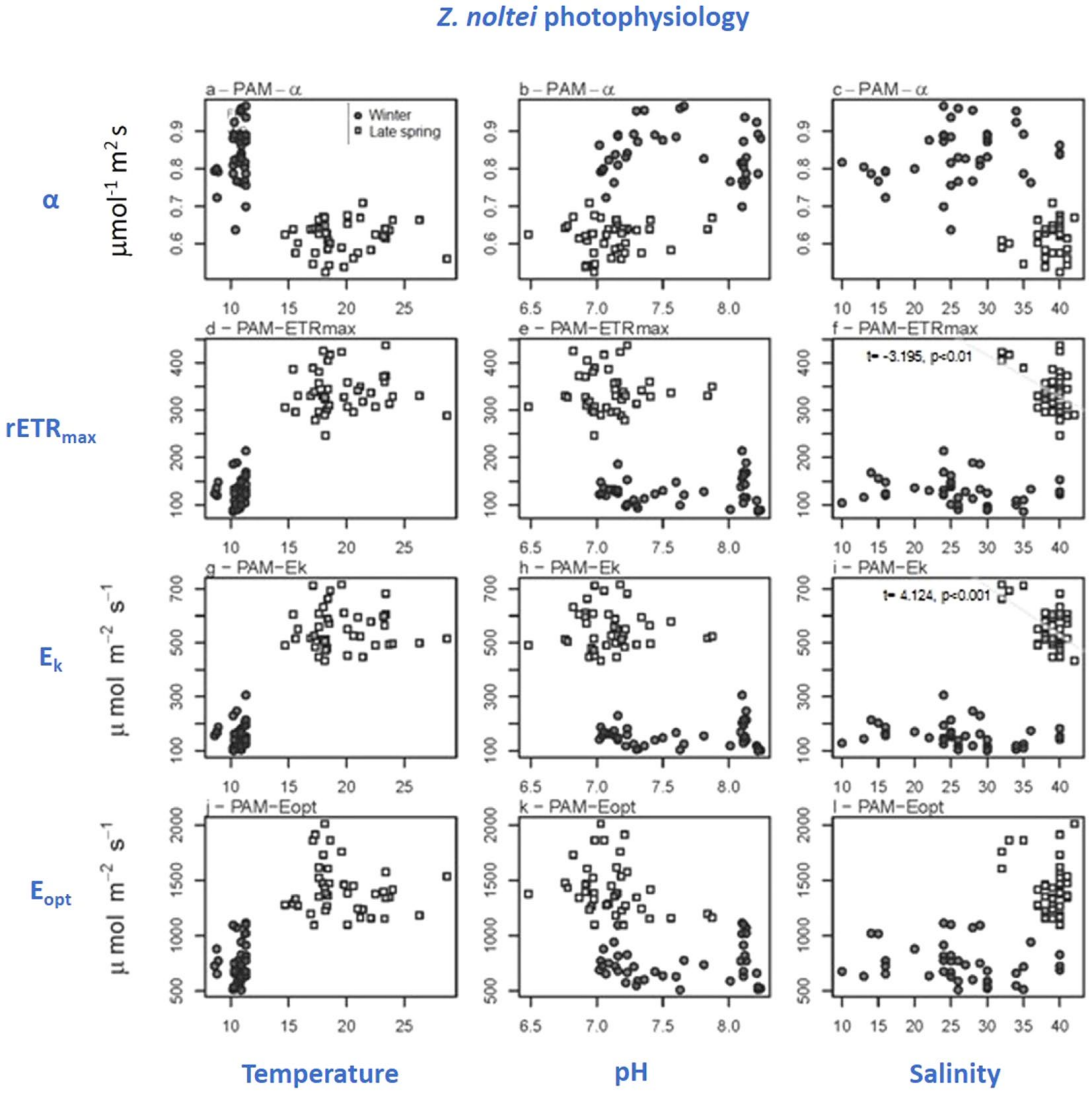
*Z*. *noltei* photosynthetic performance *versus* temperature, pH and salinity in winter and late spring. Maximum photosynthetic efficiency (α) *versus* (**a**) temperature, (**b**) pH and (**c**) salinity; maximum relative electron transport rate (rETR_m_) *versus* (**d**) temperature, (**e**) pH and (**f**) salinity; photoacclimation index (E_k_) *versus* (**g**) temperature, (**h**) pH and (**i**) salinity; optimum irradiance (E_opt_) *versus* (**j**) temperature, (**k**) pH and (**l**) salinity. Statistical significant linear relationships within each season are shown (p < 0.05). All statistical results are shown as Supplementary information (Table S1).

Within seasons, there was a significant positive relationship between temperature and aboveground biomass, carbon pool and nitrogen pool (*p* < 0.05) in winter (Fig. 4Aa,d,g; Table S1, from supplementary information); between pH and aboveground biomass (*p* < 0.05) in late spring (Fig. 4Ab); and between temperature and belowground biomass (*p* < 0.05) in winter (Fig. 4Ba; Table S1). Also, there was a significant negative relationship between salinity and maximum relative electron transport rate (rETR_m_) (*p* < 0.01) (Fig. 5f) and photoacclimation index (E_k_) (*p* < 0.001) in late spring (Fig. 5i; Table S1). On the contrary, there was no significant relationship between temperature, pH and salinity and *Zostera* belowground C pool (Fig. 4Bd,e,f; Table S1), belowground N pool (Fig. 4Bg,h,i), photosynthetic efficiency (α) (Fig. 5a,b,c), and optimum irradiance (E_opt_) (Fig. 5j,k,l) in both seasons; and no significant relationships between pH and salinity, and *Zostera* aboveground C pool (Fig. 4Ae,f), aboveground N pool (Fig. 4Ah,i), belowground biomass (Fig. 4Bb,c; Table S1), in both seasons.

### *Zostera noltei* traits versus spatio-temporal shifts in environmental parameters

**Figure 6:**
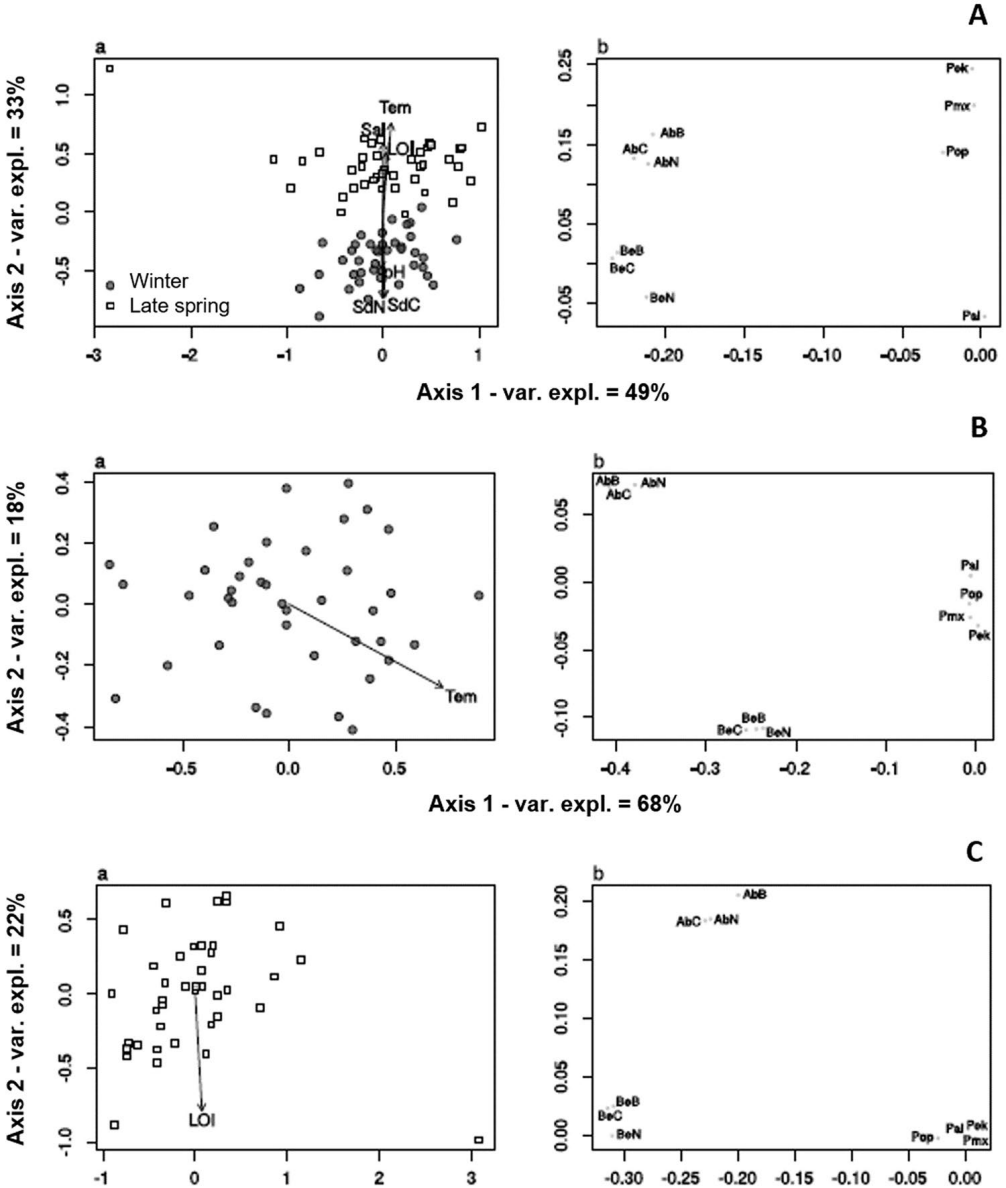
Principal Coordinates Analysis (PCO) showing the variation among sampling sites. *Z*. *noltei* traits included are: above- (AbB) and belowground biomass (BeB), above- (AbC) and belowground C pool (BeC), above- (AbN) and belowground N pool (BeN); and photosynthetic parameters (photosynthetic efficiency (α = Pal), maximum relative electron transport rate (rETR_m_ = Pmx), photoacclimation index (E_k_ = PEk) and optimum irradiance (E_opt_ = Pop). Measured environmental variables are salinity (interstitial water) (Sal), sediment LOI, pH, temperature (Tem), sediment C pool (SdC) and sediment N pool (SdN). The distance matrices contained (**A**) all data, (**B**) data from winter only and (**C**) data from late spring only.

The PCO ordination (Fig. 6A) was primarily related to variation between samples’ above- and belowground biomass, C and N pools *versus* photosynthetic traits (49% variation explained, axis 1) (Fig. 6Ab). There was no obvious relationship between this gradient and any of the measured environmental variables (Fig. 6Aa). The second PCO axis in contrast showed a clear seasonal relationship with winter samples having higher sediment C and N pools and pH. In turn, late spring samples had higher temperature, LOI and salinity (33% variation explained, axis 2) (Fig. 6Aa). *Z*. *noltei* aboveground biomass, C and N pool and photosynthetic performance parameters (rETR_m_, E_k_ and E_opt_), were higher in late spring. On contrary, belowground biomass, C and N pool, and photosynthetic efficiency (α) were similarly related to both seasons (Fig. 6A).

When considering solely the samples collected during winter (Fig. 6B), the first PCO axis (68% variation explained) was again primarily related to variation among above- and belowground biomass, C and N pools and photosynthetic parameters (Fig. 6Bb). This axis appeared to be related to sediment temperature (Fig. 6Ba) with warmer sites in winter having lower *Z*. *noltei* biomass, and lower C and N pools. The secondary axis (18% variation explained) was primarily related to variation between sites with high aboveground *versus* high belowground biomass and respective C and N pools (Fig. 6Bb). When considering only the samples taken in late spring (Fig. 6C), the first PCO axis (74% variation explained) was yet again primarily related to variation between above- and belowground biomass, and the respective C and N pools *versus* photosynthetic parameters (Fig. 6Cb). The second axis (22% variation explained) was primarily related to variation of *Z*. *noltei* aboveground biomass, C and N pool *versus* belowground biomass, C and N pool, whose higher values are related to a higher LOI (Fig. 6Ca).

### Esterified fatty acid profiles in *Zostera noltei*

Bearing in mind the main objective of the present work, the sixteen most abundant esterified fatty acids (FA) identified from *Z*. *noltei* samples were selected for further analysis. Overall, *Z*. *noltei* aboveground biomass showed a higher total FA concentration than belowground biomass, both in winter and late spring (Table S2, from supplementary information). Analysing the FA profiles (net concentration) in *Z*. *noltei* aboveground biomass, the multivariate dispersions of the FA were equal among sites (PERMDISP, F = 2.865, *p* > 0.05) and seasons (PERMDISP, F = 1.242, *p* > 0.05). On the contrary, significant differences were recorded for FA profiles on belowground biomass among sites (PERMDISP, F = 6.993, *p* < 0.01). *Z*. *noltei* from site 10 showed a FA composition statistically different from all the other sites (1, 2, 5 and 7). These differences are mainly explained by the FA 16:1*n*-7, 20:4*n*-6, 20:5*n*-3, 22:6*n*-3 and 24:1*n*-9 (SIMPER analysis; Table S3, from supplementary information). However, the mentioned FA altogether account for a very small proportion (1.0 ± 0.5% in late spring to 5.0 ± 1.1% in winter) of total FA in the belowground biomass. Since there were no differences in the FA profiles among these sites, relevant differences are not expected to occur among the other five sampling sites (3, 4, 6, 8 and 9). Therefore, the latter sites were not analysed for FA profiles.

### Esterified fatty acids: SFA, MUFA and PUFA

**Figure 7:**
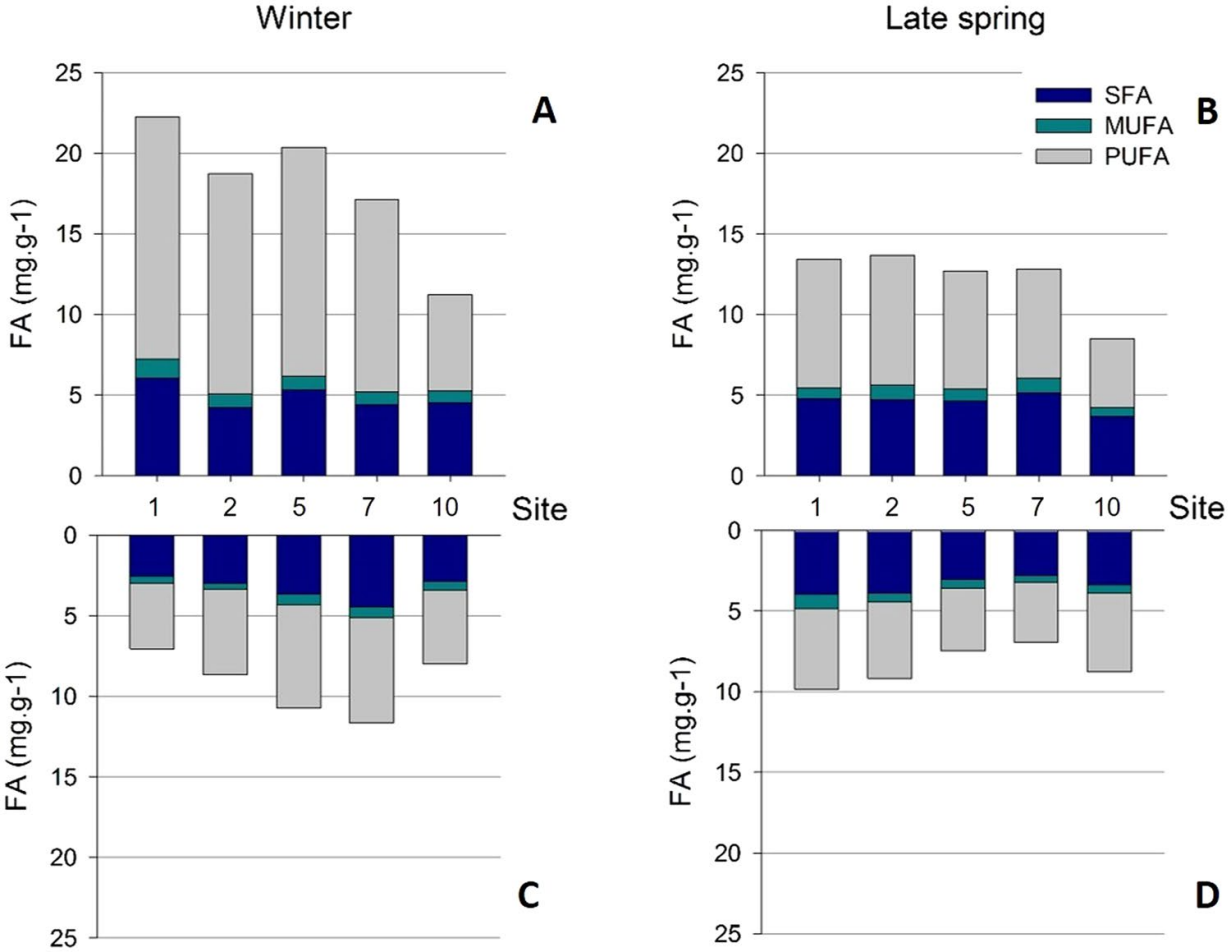
Esterified fatty acid (FA) concentration (mg g^−1^) in *Z*. *noltei* aboveground biomass at sites 1, 2, 5, 7 and 10 at Mira channel; in winter (**A**) and late spring (**B**). Graphs (**C**) and (**D**) show the FA concentration in belowground biomass at the same sites, for winter and late spring, respectively. Saturated fatty acids (SFA), monounsaturated fatty acids (MUFA) and polyunsaturated fatty acids (PUFA) concentrations are shown at all graphs (mean values, N = 3).

Once grouping FA in saturated fatty acids (SFA), monounsaturated fatty acids (MUFA) and polyunsaturated fatty acids (PUFA), no differences on the dispersions were found for *Z*. *noltei* aboveground biomass between seasons (winter *vs* late spring) (PERMDISP, F = 0.303, *p* > 0.05) (Fig. 7). Also, no differences were recorded among sites along Mira channel (PERMDISP, F = 1.032, *p* > 0.05) (Fig. 7). The same result was obtained for belowground biomass, meaning that SFA, MUFA and PUFA profiles did not differ significantly among season (PERMDISP, F = 0.245, *p* > 0.05) nor among sites (PERMDISP, F = 2.241, *p* > 0.05). Globally, PUFA relative contribution to the total FA profile was higher than the contribution of SFA and MUFA altogether (Fig. 7; Table S2, from supplementary information). Namely, PUFA accounted for 52–73% (mean 66 ± 8%) of the total FA in winter and 56–61% in late spring (mean 58 ± 2%) in *Z*. *noltei* aboveground biomass. In belowground biomass, PUFA accounted for 49–59% (mean 55 ± 4%) in winter and 52–56% (mean 53 ± 2%) in late spring. Thus, PUFA mean FA fraction in aboveground biomass was slightly higher than in belowground biomass. The dominant PUFA of *Z*. *noltei* were linoleic (18:2*n*-6) and alpha linolenic acid (18:3*n*-3), both being seagrass (or vascular plants) markers; and 20:4, 20:5 and 22:6 accounting for only <2% of total PUFA, both in above- and belowground *Z*. *noltei* biomass (Table S2, from supplementary information). Relative contribution of SFA was 29–36% of total FA in aboveground biomass and about 40% in belowground biomass, with palmitic acid (16:0), an ubiquitous FA, as the major SFA (73–78% of total SFA). The proportion of MUFA was considerably lower, being about 5–6% of total FA in aboveground biomass and about 7% in belowground *Z*. *noltei* biomass.

**Figure 8:**
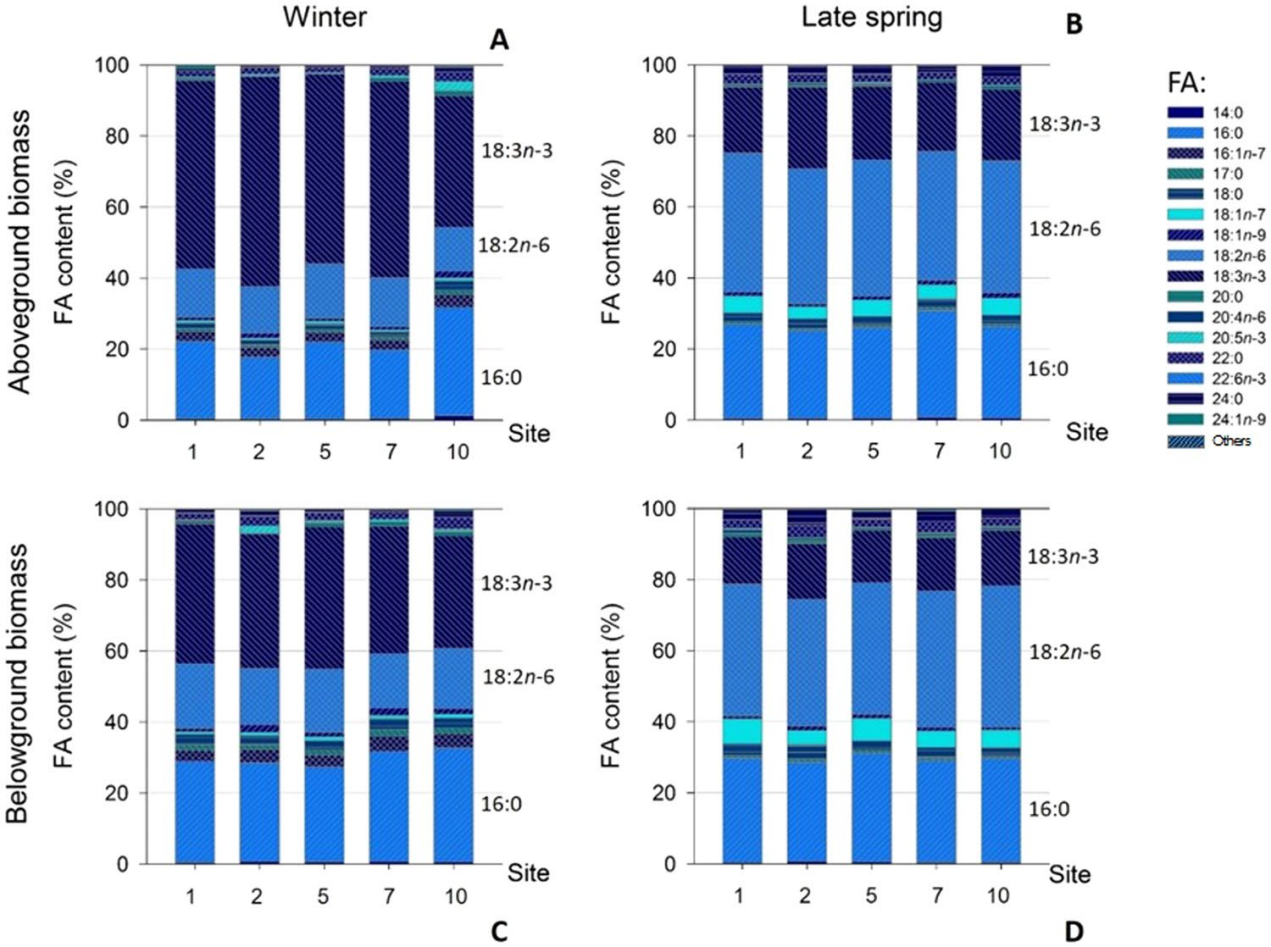
Individual esterified fatty acid (FA) relative content (%) in *Z*. *noltei* aboveground biomass in winter (**A**) and late spring (**B**) along Mira channel. FA relative content in belowground biomass are shown also for winter (**C**) and late spring (**D**) (mean values, N = 3). “Others” refer to the sum of FA 10:0, 11:0, 12:0, 13:0, 14:1, 15:0, 15:1, 16:1*n*-9, 19:3, 20:1, 20:2, 20:3*n*-3, 20:3*n*-6 and 23:0.

Concerning the two seagrass PUFA markers, linoleic acid (18:2*n*-6) showed similar relative content in *Z*. *noltei* above- and belowground biomass in winter (14 ± 1 and 17 ± 1% of total FA, respectively), and increased to 38 ± 2% of total FA in late spring (Fig. 8). On the contrary, alpha linolenic acid (18:3*n*-3) showed higher relative content in winter (52 ± 8% of total FA in aboveground and 37 ± 3% in belowground biomass). In late spring, alpha linolenic acid content decreased to 20 ± 2 and 15 ± 1% of total FA, for aboveground and belowground biomass, respectively. Both linoleic and alpha linolenic acid showed a similar relative content among studied sites in winter and late spring.

## Discussion

*Z*. *noltei* meadows were shown to be, but merely to some extent, affected by their habitat spatial dimensions, namely spatial shifts of salinity, sediment temperature and pH. In late spring, spatial shifts of salinity (ranging from 33 to 41), negatively affected *Zostera* photosynthetic performance, particularly _r_ETR_m_ (related to the maximum photosynthetic capacity) and E_k_ (photoacclimation index). However, also in late spring, other photosynthetic parameters (α and E_k_) were not affected by spatial salinity differences. Consequently, it reduces the range and impact of negative salinity effects on the *Z*. *noltei* photosynthetic performance. In winter, even though a larger salinity range was recorded (from 14 to 39), *Z*. *noltei* photosynthetic performance was not affected by this spatial salinity shift. Thus, our hypothesis (H_0_1), stating that high salinity does not affect photosynthetic performance is only to some extent corroborated.

Less important was the positive relationship of sediment temperature in winter (ranging from 12.2 to 15.6) and *Z*. *noltei* biomass, aboveground C and N pool. The positive relationship of pH spatial shift (from 6.9 to 7.5) and aboveground biomass in late spring was also less relevant. Besides these effects, most of the *Z*. *noltei* traits were not significantly related to spatial shifts in these environmental parameters. Therefore, overall, spatial shifts in salinity, temperature or pH do not (totally) explain differences on *Z*. *noltei* ecological processes. Only photosynthetic performance and biomass can be partially explained, and not during both seasons surveyed (winter and late spring).

Seasonal changes in salinity and other environmental parameters (sediment pH, temperature, LOI and C and N pools) explain the observed seasonal differences in *Z*. *noltei* ecological processes and functions. Its aboveground biomass, C and N pools, and photosynthetic performance demonstrate this. Therefore, the following ecosystem services: 1) biomass provisioning (through primary production), 2) nutrient cycling (through N uptake and storage) and 3) climate regulation (through C fixation and storage) might change seasonally but are not disrupted (meaning that H_0_2 can be accepted).

Essentially, *Z*. *noltei* meadows have an important role in biomass provisioning through photosynthesis and in nutrient cycling, namely through the uptake of inorganic N and its incorporation in biomass^45^. The organic matter (including organic N) burial in sediment, which will later be mineralized, is also important in N cycling. In addition, seagrass meadows are known to be important C sinks^3^. Namely, *Z*. *noltei* belowground biomass plays an important role in carbon storage. *Z*. *noltei* C sequestration through incorporation of atmospheric CO_2_ (photosynthesis) and its investment in biomass (C storage)^7^ are important ecological process. In turn, these processes contribute to climate regulation (ES), by the reduction of greenhouse gas concentrations. Additionally, *Z*. *noltei* itself has a mass stabilizing effect (ES), being able to reduce sediment resuspension and erosion rates, minimize current velocity and increase the light availability in water column^4, 5^.

*Z*. *noltei* aboveground and belowground biomass recorded here is within the range found for this species in other geographical locations (Table 2). The above:belowground biomass ratio showed a similar trend (>1) to that previously described for Mondego estuary (Portugal)^46^, Ria Formosa (Portugal)^21, 47^ and for meadows at Palmones River estuary (Spain)^48^ (Table 2). In contrast, this ratio was <1 at Ischia, Gulf of Naples (Italy)^49^. In late spring, *Z*. *noltei* greatly invested in the aboveground biomass (significantly higher than in winter), which is frequently explained by favourable environmental conditions (higher temperatures and light availability)^48^. In turn, investment in belowground biomass has been attributed to an acclimation strategy of *Z*. *noltei* to environmental stress conditions, namely high hydrodynamics alone^50^ or combined with saturating light conditions^51^. In our study site (Mira channel, Ria Aveiro), *Z*. *noltei* does not appear to be under hydrodynamic stress, which is in line with water current velocities estimated for the study area (0.3 m s^−1^ at the channel head)^52^.

**Table 2:** *Zostera noltei* biomass, carbon (C) and nitrogen (N) dynamics at different geographical locations (AG – aboveground; BG – belowground; mean ± standard deviation; DW: dry weight).

Location	Biomass (gDW m^−2^) (mean ± SD)	AG:BG biomass ratio	C:N ratio (mean ± SD)	C content (% DW) (mean ± SD)	N content (% DW) (mean ± SD)	C pool (gC m^−2^) (mean ± SD)	N pool (gN m^−2^) (mean ± SD)	References
Ria Aveiro (Portugal)	AG: - winter: 76 ± 28 - late spring: 106 ± 49 - mean: 91 ± 42 ﻿BG: - winter: 43 ± 10 - late spring: 49 ± 18 - mean: 46 ± 15	>1	AG: - winter: 9.4 ± 0.5 - late spring: 9.9 ± 0.9 -mean: 9.7 ± 0.7 ﻿BG: - winter: 18.2 ± 1.0 - late spring: 23.5 ± 4.1 - mean: 20.9 ± 3.9	AG: - winter: 37 ± 1 - late spring: 37 ± 1 - mean: 37 ± 1 ﻿BG: - winter: 33 ± 2 - late spring: 32 ± 1 - mean: 33 ± 1	AG: - winter: 3.9 ± 0.2 - late spring: 3.7 ± 0.3 - mean: 3.8 ± 0.3 ﻿BG: - winter: 1.8 ± 0.1 - late spring: 1.4 ± 0.2 - mean: 1.6 ± 0.3 ﻿AG + BG: -﻿ 2.7﻿	AG: - winter: 28 ± 10 - late spring: 39 ± 18 - mean: 33 ± 15 ﻿BG: - winter: 14 ± 4 - late spring: 16 ± 6 - mean: 15 ± 5	AG: - winter: 2.9 ± 1.0 - late spring: 3.9 ± 1.8 - mean: 3.4 ± 1.5 ﻿BG: - winter: 0.8 ± 0.2 - late spring: 0.7 ± 0.3 - mean: 0.7 ± 0.3	This study
	AG + BG (g AFDW.m^−2^): - 86 to 170 (min-max) - mean: 110 ± 50	—	—	—	AG + BG: - 2.09 ± 0.48	—	AG + BG: 3.8 ± 1.7	54
Mondego estuary (Portugal)	AG: (gAFDW.m^−2^) - 0.1–198 (min-max) - mean ± SE:55 ± 4 ﻿BG: (gAFDW.m^−2^) - 2 to 67 (min-max) - mean ± SE:28 ± 1	>1	—	—	AG: - 0.6 to 4.5 (min-max), - mean ± SE: 3.0 ± 0.1 ﻿BG: -﻿0.3 to 4.7 (min-max) - 1.9 ± 0.2 (mean ± SE)	—	—	46
Ria Formosa (Portugal)	AG (min-max): - low intertidal: 47 to 442 - mid int.: 82 to 329 - high int.: 85 to 184 ﻿BG (min-max): - low int.: 63 to 199 - mid int.: 103 to 215 - high int.: 44–140	>1	AG: - low int.: 13.1 - mid int.: 12.0 - high int.: 11.2	AG: - low int.: 43 ± 0 - mid int.: 44 ± 1 - high int.: 43 ± 1	AG: - low int.: 3.3 ± 0.0 - mid int.: 3.6 ± 0.1 - high int.: 3.8 ± 0.1 - ca. 1.5 to 4.75 (min-max) ^(1)^﻿ BG ^(1)^: - ca. 0.75 to 2.5 (min-max)	—	—	20, 21, 47
Palmones River estuary (Spain)	AG: - 30–50 to 180–200 ﻿BG: - 25–30 to 70–75 (min. winter to max. summer)	>1	AG: - 9.9 to 10 (min-max; summer-winter) ﻿BG: - 10.5 to 18 (min-max; summer-winter)	AG: - ca. 30–40 (min-max) BG: - 25 to 40 (min-max)	AG: - 3.0 to 4.0 BG: -1.2 to 3.0 (min-max; summer-winter)	AG: - ca. 10– 67 (min- max) - mean: 37.1 BG: - ca. 5–30 (min-max) - mean: 16.2	AG: - ca. 1–6 (max-min) - mean: 3.5 BG: - ca. 0.5–2 (min-max) - mean: 1.5	48
Ischia, Gulf of Naples (Italy)	AG: - ca. 8–33 BG: - ca. 16–45	<1	—	—	AG: - ca. 1.3 to 2.75 (min-max) BG: - ca. 1.3 to 2.6 (min-max)	—	AG: - 0.1 to 0.5 (min-max) BG: - 0.15 to 0.33 (min-max)	49

In the current work, the C:N ratio in belowground material of *Z*. *noltei* increased from winter to late spring as a result of a reduction in the N pool, which is in line with data from the Palmones River estuary (Spain) and Gulf of Naples (Italy) (Table 2). The maximum N content in *Z*. *noltei* belowground biomass during winter is likely due to a storage strategy for N that may be used in the following months. Along the surveyed spatial-temporal shifts of salinity at Mira channel, the N content of aboveground biomass recorded in both seasons was always higher than the nutrient limitation threshold for seagrass shoots (1.8%^53^).

The N content of *Z*. *noltei* in the Mira channel in winter and spring is similar to that recorded at Ria Formosa (Portugal), respectively in aboveground biomass^21^ and in belowground biomass^20^ (Table 2). Moreover, it is similar to that found in the Mondego estuary (Portugal) in above- and belowground biomass^46^, but higher than the N content recorded by Peralta *et al*.^20^ for aboveground biomass. Figueiredo da Silva *et al*.^54^ previously showed a mean N content of 2% (%DW) in the Ria de Aveiro, whereas our study revealed a mean *Z*. *noltei* N content of 2.7% (mean of above- and belowground biomass N content in winter and late spring) (Table 2). The C pool of *Z*. *noltei* aboveground biomass is in the same order of magnitude of previous studies^48^, but higher than values reported for both above- and belowground biomass by Peralta *et al*.^55^.

*Z*. *noltei* has been shown to be able to adapt its biomass partitioning (above:belowground ratio) and morphology to environmental pressure^56^. Indeed, its plasticity was demonstrated at different organizational levels (cell, individual and population) along an intertidal vertical gradient in the Ria Formosa, reinforcing its ability to acclimate to abrupt environmental gradients^21^.

The photosynthetic performance of *Z*. *noltei*, particularly concerning light-saturated photosynthesis (rETR_m_), is in line with values reported in the literature^57, 58^, suggesting that the specimens surveyed were adapted to the physicochemical conditions along the channel. The photosynthetic performance of *Z*. *noltei* revealed a seasonal pattern in photoacclimation state, characterized by an overall optimization of light usage through change in both light-limited and light-saturated photosynthetic activity. Higher light absorption efficiency (α) was observed in winter, allowing *Z*. *noltei* to cope with the lower light availability. On the other hand, rETR_m_ increased towards summer, denoting an increased efficiency of the carbon fixation metabolism, with *Z*. *noltei* taking advantage of higher light levels and temperature. However, as mentioned before, maximum relative electron transport rate (rETR_m_) and photoacclimation index (E_k_) were negatively affected by salinity increase in late spring. Of interest, this seasonal change occurred irrespectively of the surveyed spatial shifts of salinity. In winter ranged from 14 to 39; in late spring, ranged from 33 to 41; i.e., essentially the same pattern in all sampling sites. No trend was evidenced along the surveyed spatial shifts of salinity at Mira channel.

A previous experimental study by Fernández-Torquemada and Sánchez-Lizaso^22^, showed that *Z*. *noltei* (in Alicante, SE Spain) is more tolerant to lower salinities (e.g., 2) than to hypersalinity (shoot mortality significantly increased at salinities >47). Moreover, it has been shown *in situ* (Vaccarès lagoon, Southern France) that *Z*. *noltei* is not outcompeted by other species, even under long-term exposure to low salinities (e.g., exposed to a salinity of 5 for 3 years)^58^. In laboratorial experiments, *Z*. *noltei* germination rate and seedling survival was shown to be higher at low salinity (1) and decrease with increasing salinities (10 to 40)^59^. Overall, the tolerance shown by *Z*. *noltei* to spatio-temporal shifts of salinity (ranging between 14 and 41 in interstitial water) may play a key role in its resilience to extreme weather events. Indeed, these events are likely to affect the freshwater flows into this coastal lagoon. Considering the cited works, *Z*. *noltei* is likely to survive and adapt to salinity changes as a result of extreme droughts or flood events. Thus, the studied ecological processes are not expected to be at risk.

Absence of relevant differences on the *Z*. *noltei* individual FA profile along the Mira channel and between seasons (winter and late spring) corroborates the plasticity mentioned above for this seagrass, in face of the spatial salinity shifts and the seasonal environmental changes recorded. Only *Z*. *noltei* belowground biomass from site 10 (the most upstream location surveyed) showed a contrasting FA profile from those recorded in other sampling sites, which was explained by the FA 16:1*n*-7, 20:4*n*-6, 20:5*n*-3, 22:6*n*-3 and 24:1*n*-9. However, consistent with previous results for *Z*. *noltei* ^30, 60, 61^, these FA relative content correspond to a very small proportion of total FA (≤5%, altogether). Moreover, palmitoleic (16:1*n*-7) and eicosapentaenoic acid (20:5*n*-3) are frequently used as diatom markers, while docosahexaenoic acid (22:6*n*-3) is a well-established marker for flagellates^62^. Therefore, these FA may likely origin from diatoms and flagellates neighbouring *Z*. *noltei*. It reveals that the difference recorded on the FA profile may not be related to seagrass physiology.

FA composition in seagrasses^63^ and seaweeds^27^ is known to depend on environmental parameters such as light^28^, temperature^26^, salinity^25^ or nutrient availability^64^. Actually, considering that marine macrophytes are ectothermic organisms, membrane fluidity depends on the environmental temperature^65^ and seasonal changes in FA composition can occur^26, 63^. However, in the present work and supported by the results obtained for the analyses of individual FA profiles, no significant differences were recorded in the *Z*. *noltei* FA profiles, when they are grouped as SFA, MUFA and PUFA. Namely, sediment temperature increased from 14 ± 1 °C in winter (mean ± standard deviation, all sites) to 19.3 ± 2.9 °C in late spring, but it did not trigger a significant change on *Z*. *noltei* FA profile. Even though net PUFA in aboveground biomass was to some extent higher in winter (12.1 ± 3.6 mg g^−1^, mean ± standard deviation, all sites) than in late spring (6.9 ± 1.6 mg g^−1^), evidencing a slight adaptation of *Z*. *noltei* to the environmental changes recorded, this difference was not statistically significant. In turn, acclimation of *Z*. *marina* to temperature through changes in FA composition was reported by Sanina *et al*.^63^ in the Sea of Japan, when water temperature decreased from 20–23 °C (summer) to 3 °C (winter). *Z*. *marina* PUFA/SFA ratio increased from summer to winter due to an increase on *n*-3/*n*-6 PUFA’s ratio. In tropical brown seaweeds, lower temperatures were also shown to promote a decrease in SFA and an increase in PUFA, so that membrane fluidity is maintained even at lower temperatures^27^. In addition, considering that salt stress in photosynthetic organisms can be perceived through FA desaturation^66^, the spatial and temporal salinity shifts (from 14 to 41) observed did not affect *Z*. *noltei* FA synthesis and is therefore easily endured by this seagrass. Overall, environmental changes recorded at Mira channel were not sufficient to trigger and significantly modify the FA composition in *Z*. *noltei* (therefore accepting our hypothesis H_0_3). Once again, the obtained results corroborate the resilience of this species to environmental fluctuations.

Major FA profiles (relative content) recorded in this work are in line with previous works performed for *Z*. *noltei* at different geographical regions such as Ria de Aveiro^60^ and Ria Formosa^61^, in Portugal and Marennes-Oléron Bay, in the French Atlantic coast^30^. Also, the high PUFA relative content in *Z*. *noltei* biomass, higher than the sum of all SFA and MUFA present in the FA pool, is in accordance with the above mentioned studies performed on *Z*. *noltei* at Ria de Aveiro and Ria Formosa, as well as data recorded for *Z*. *marina* at Ria Formosa^61^ and Sea of Japan^63^.

Besides the environmental variables addressed in the current work, other environmental variables and stressors can affect the *Z*. *noltei* ecological processes studied. For instance, light availability^55, 67^ was shown to influence the overall *Z*. *noltei* growth, but also the plant development pattern, such as growth of lateral shoots from the main rhizomes^55^. In addition, under increasing light levels, the nitrogen content decreases, particularly in belowground material^55^. Nevertheless, the elevation of *Z*. *noltei* sampling sites along Mira channel is similar, meaning that daily hours of emersion (and immersion) for this intertidal seagrass is similar at all sites^68^. Therefore, the effect of light availability on *Z*. *noltei* ecological processes is likely to be the same at all sampling sites.

Overall, shifts in salinity and other environmental parameters recorded at Mira channel did not trigger changes in the *Z*. *noltei* FA profile, which corroborates the resilience potential of this seagrass to salinity shifts, which are likely to increase in the future.

Current climate change scenarios projected for this system predict an increase in flood area due to mean sea level rise, with Mira channel being likely to experience erosion that may lead to a shoreline retreat, a reduction in sand spit stability and even to a potential opening of a new inlet upstream (and consequent seawater intrusion)^69^. In addition to this, projected river discharges from the catchment to the lagoon, to the mid and end of this century, predict a reduction in average annual water discharges, with freshwater contributions to the lagoon decreasing, especially during summer^70^. Even though the predicted increase in flood area and the potential for seawater intrusion in Ria de Aveiro, the plasticity observed for *Z*. *noltei* – at different levels – to salinity shifts may anticipate its ability to cope with those changes, as well to expand to adjacent unvegetated areas in the Mira channel.

## Conclusions

*Z*. *noltei* proved to be euryhaline, with its biology being only slightly affected within the salinity shifts surveyed in each season (i.e. between 14 and 39 in winter; and 33 and 41 in late spring). Seasonal differences in *Z*. *noltei* traits, such as aboveground biomass, aboveground N and C pools, as well as photosynthetic performance, are explained by the pronounced seasonal shifts in salinity and other environmental parameters (temperature, pH, LOI, sediment C and N pool). Fatty acid synthesis in *Z*. *noltei* was not affected by the spatio-temporal environmental fluctuations recorded. To sum up, our results show that, within the salinity range surveyed, the ecological processes addressed in our study supporting the ecosystem services provided by *Z*. *noltei* meadows do not appear to be at risk. Considering the wide geographical distribution of this seagrass (eastern Atlantic coastline) and the global predictions of increasing extreme weather events, these findings can anticipate the plastic response of the considered traits of *Z*. *noltei* to shifts in salinity and potential impacts on the ES provided.

## Electronic supplementary material

Supplementary information

## References

[1] Short FT, Carruthers TJB, Dennison WC, Waycott M (2007). Global seagrass distribution and diversity: a bioregional model. Journal of Experimental Marine Biology and Ecology.

[2] Cullen-Unsworth L, Unsworth R (2013). Seagrass Meadows, Ecosystem Services, and Sustainability. Environment. Science and Policy for Sustainable Development.

[3] Duarte CM, Losada IJ, Hendriks IE, Mazarrasa I, Marbà N (2013). The role of coastal plant communities for climate change mitigation and adaptation. Nature Climate Change.

[4] Wilkie L, O’Hare MT, Davidson I, Dudley B, Paterson DM (2012). Particle trapping and retention by *Zostera noltii*: a flume and field study. Aquatic Botany.

[5] Cunha AH, Assis JF, Serrão EA (2013). Seagrasses in Portugal: A most endangered marine habitat. Aquatic Botany.

[6] Delgard M-L (2013). Changes in Nutrient Biogeochemistry in Response to the Regression of *Zostera noltii* Meadows in the Arcachon Bay (France). Aquatic Geochemistry.

[7] Clavier J (2011). Aerial and underwater carbon metabolism of a *Zostera noltii* seagrass bed in the Banc d’Arguin, Mauritania. Aquatic Botany.

[8] Luisetti T, Jackson EL, Turner RK (2013). Valuing the European ‘coastal blue carbon’ storage benefit. Marine Pollution Bulletin.

[9] Jackson EL, Rees SE, Wilding C, Attrill MJ (2015). Use of a seagrass residency index to apportion commercial fishery landing values and recreation fisheries expenditure to seagrass habitat service. Conservation Biology.

[10] Waycott M (2009). Accelerating loss of seagrasses across the globe threatens coastal ecosystems. Proceedings National Academic of Science.

[11] Short FT (2011). Extinction risk assessment of the world’s seagrass species. Biological Conservation.

[12] Lillebø, A.I. *et al*. Restoration of Seagrass Community to Reverse Eutrophication in Estuaries. In Wolanski, E. and McLusky, D.S. (Eds) Treatise on Estuarine and Coastal Science. Elsevier Waltham, Academic Press, pp. 151–164 (2011).

[13] Valdemarsen T (2010). Vulnerability of *Zostera marina* seedlings to physical stress. Marine Ecology Progress Series.

[14] Serrano O (2016). Impact of mooring activities on carbon stocks in seagrass meadows. Sci. Rep..

[15] Marbà N, Duarte CM (2010). Mediterranean warming triggers seagrass (*Posidonia oceanica*) shoot mortality. Global Change Biology.

[16] Plus MD (2010). Long-term evolution (1988–2008) of *Zostera* spp. meadows in Arcachon Bay (Bay of Biscay). Estuarine, Coastal and Shelf. Sciences.

[17] Green, E. P. & F. T., Short. *World Atlas of Seagrasses*. University of California Press, Los Angeles, 298 pp (2003).

[18] Vermaat JE, Verhagen FCA, Lindenburg D (2000). Contrasting responses in two populations of *Zostera noltii* Hornem. to experimental photoperiod manipulation at two salinities. Aquatic Botany.

[19] den Hartog, C. *The seagrasses of the world*. North-Holland Publ., Amsterdam, 275 (1970).

[20] Peralta G, Brun FG, Hernandez I, Vergara JJ, Pérez-Lloréns JL (2005). Morphometric variations as acclimation mechanisms in *Zostera noltii* beds. Estuarine, Coastal and Shelf Science.

[21] Cabaço S, Machás R, Santos R (2009). Individual and population plasticity of the seagrass *Zostera noltii* along a vertical intertidal gradient. Estuarine, Coastal and Shelf Science.

[22] Fernández-Torquemada Y, Sánchez-Lizaso JL (2011). Responses of two Mediterranean seagrasses to experimental changes in salinity. Hydrobiologia.

[23] Touchette BW (2007). Seagrass-salinity interactions: physiological mechanisms used by submersed marine angiosperms for a life at sea. Journal of Experimental Marine Biology and Ecology.

[24] Sandoval-Gil JM, Marín-Guirao L, Ruiz JM (2012). Tolerance of Mediterranean seagrasses (*Posidonia oceanica* and *Cymodocea nodosa*) to hypersaline stress: water relations and osmolyte concentrations. Marine Biology.

[25] Kumar M, Kumari P, Gupta V, Reddy C, Jha B (2010). Biochemical responses of red alga *Gracilaria corticata* (Gracilariales, Rhodophyta) to salinity induced oxidative stress. Journal of Experimental Marine Biology and Ecology.

[26] Becker S, Graeve M, Bischof K (2010). Photosynthesis and lipid composition of the Antarctic endemic rhodophyte Palmaria decipiens: effects of changing light and temperature levels. Polar Biol.

[27] Nomura M (2013). Seasonal variations of total lipids, fatty acid composition, and fucoxanthin contents of *Sargassum horneri* (Turner) and *Cystoseira hakodatensis* (Yendo) from the northern seashore of Japan. Journal of Applied Phycology.

[28] Hotimchenko SV (2002). Fatty acid composition of algae from habitats with varying amounts of illumination. Russian J Plant Physiol.

[29] Kharlamenko VI, Kiyashko SI, Imbs AB, Vyshkvartzev DI (2001). Identification of food sources of invertebrates from the seagrass *Zostera marina* community using carbon and sulfur stable isotope ratio and fatty acid analyses. Marine Ecology Progress Series.

[30] Lebreton B (2011). Trophic importance of diatoms in an intertidal Zostera noltii seagrass bed: Evidence from stable isotope and fatty acid analyses. Estuarine, Coastal and Shelf Science.

[31] Hootsmans MJM, Vermaat JE, van Vierssen W (1987). Seed bank development, germination and early seedling survival of two seagrass species from The Netherlands: *Zostera marina* L. and *Zostera noltti* Hornem. Aquatic Botany.

[32] MAMAOT/ARHCentro. Plano de Gestão das Bacias Hidrográficas dos rios Vouga, Mondego e Lis Integrados na Região Hidrográfica 4, Parte 4 – Cenários prospectivos, 4 – Impactes sectoriais das Alterações Climáticas. *Ministério da Agricultura, Mar, Ambiente e Ordenamento do Território/Administração da Região Hidrográfica do Centro I.P* pp.13 (2012).

[33] International Panel Climate Change: Impacts, Adaptation, and Vulnerability. *Part A: Global and Sectoral Aspects. Contribution of Working Group II to the Fifth Assessment Report of the Intergovernmental Panel on Climate Change* [Field, C.B. *et al*. (eds)]. Cambridge University Press, Cambridge, United Kingdom and New York, NY, USA (2014).

[34] Moreira MH, Queiroga H, Machado MM, Cunha MR (1993). Environmental gradients in a southern estuarine ecosystem: Ria de Aveiro, Portugal. Implication for soft bottom macrofauna colonization. Netherlands. Journal of Aquatic Ecology.

[35] Rodrigues M, Oliveira A, Queiroga H, Brotas V (2012). Seasonal and diurnal water quality and ecological dynamics along a salinity gradient (Mira channel, Aveiro lagoon, Portugal). Procedia Environmental Sciences.

[36] Leandro SM, Tiselius P, Queiroga H (2013). Spatial and temporal scales of environmental forcing of *Acartia* populations (Copepoda: Calanoida) in the Canal de Mira (Ria de Aveiro, Portugal). ICES Journal of Marine Science.

[37] Dahl E (1956). Ecological Salinity Boundaries in Poikilohaline Waters. Oikos.

[38] Blott SJ, Pye K (2012). Particle size scales and classification of sediment types based on particle size distributions: Review and recommended procedures. Sedimentology.

[39] Serôdio J (2004). Analysis of variable chlorophyll fluorescence on microphytobenthos assemblages: implications of the use of depth-integrated measurements. Aquatic Microbial Ecology.

[40] Genty B, Briantais JM, Baker NR (1989). The relationship between the quantum yield of photosynthetic electron transport and quenching of chlorophyll fluorescence. Biochimica et Biophysica Acta.

[41] Schreiber U, Hormann H, Neubauer C, Klughammer C (1995). Assessment of photosystem quantum yield by chlorophyll fluorescence quenching analysis. Aust J Plant Physiol.

[42] Eilers PHC, Peeters JCH (1988). A model for the relationship between light intensity and the rate of photosynthesis in phytoplankton. Ecological Modelling.

[43] Aued-Pimentel S, Lago JHG, Chaves MH, Kumagai EE (2004). Evaluation of a methylation procedure to determine cyclopropenoids fatty acids from Sterculia striata St. Hil. Et Nauds seed oil. J. Chromatogr. A.

[44] Oksanen J (2009). vegan: Community ecology package. R package version.

[45] Alexandre A, Silva J, Bouma TJ, Santos R (2011). Inorganic nitrogen uptake kinetics and whole-plant nitrogen budget in the seagrass *Zostera noltii*. Journal of Experimental Marine Biology and Ecology.

[46] Leston S, Lillebø AI, Pardal MA (2008). The response of primary producers’ assemblages to the mitigation measures to reduce eutrophication symptoms in a temperate estuary. Estuarine, Coastal and Shelf Science.

[47] Cabaço S, Santos R (2012). Seagrass reproductive effort as an ecological indicator of disturbance. Ecological Indicators.

[48] Pérez-Lloréns JL, Niell FX (1993). Temperature and emergence on the net photosynthesis of two *Zostera noltii* Hornem. morphotypes. Hydrobiologia.

[49] Kraemer GP, Mazzella L (1999). Nitrogen acquisition, storage, and use by the Mediterranean seagrasses *Cymodocea nodosa* and *Zostera noltii*. Marine Ecology Progress Series.

[50] Peralta G, Brun FG, Pérez-Lloréns JL, Bouma TJ (2006). Direct effects of current velocity on the growth, morphometry and architecture of seagrasses: a case study on *Zostera noltii*. Marine Ecology Progress Series.

[51] de los Santos CB, Brun FG, Bouma TJ, Vergara JJ, Pérez-Lloréns JJ (2010). Acclimation of seagrass *Zostera noltii* to co-occurring hydrodynamic and light stresses. Marine Ecology Progress Series.

[52] Picado, A., Lopes, C. L., Mendes, R., Vaz, N. & Dias, J. M. *Storm surge impact in the hydrodynamics of a tidal lagoon: the case of Ria de Aveiro*. In Conley, D.C., Masselink, G., Russell, P. E. and O’Hare, T. J. (eds). *Proceedings 12th International Coastal Symposium (Plymouth, England), Journal of Coastal Research* SI 65, 796-801, ISSN 0749-0208 (2013).

[53] Duarte CM (1990). Seagrass nutrient content. Marine Ecology Progress Series.

[54] Figueiredo da Silva J, Duck RW, Catarino JB (2009). Nutrient retention in the sediments and the submerged aquatic vegetation of the coastal lagoon of the Ria de Aveiro, Portugal. Journal of Sea Research.

[55] Peralta G, Pérez-Lloréns JL, Hernandez I, Vergara JJ (2002). Effects of light availability on growth, architecture and nutrient content of the seagrass *Zostera noltii* Hornem. Journal of Experimental Marine Biology and Ecology.

[56] Olivé I, Brun FG, Vergara JJ, Pérez-Lloréns JL (2007). Effects of light and biomass partitioning on growth, photosynthesis and carbohydrate content of the seagrass *Zostera noltii* Hornem. Journal of Experimental Marine Biology and Ecology.

[57] Silva J, Santos R (2004). Can chlorophyll fluorescence be used to estimate photosynthetic production in the seagrass *Zostera noltii*?. Journal of Experimental Marine Biology and Ecology.

[58] Charpentier A, Grillas P, Lescuyer F, Coulet E, Auby I (2005). Spatio-temporal dynamics of a *Zostera noltii* dominated community over a period of fluctuating salinity in a shallow lagoon, Southern France. Estuarine, Coastal and Shelf Science.

[59] Hootsmans MJM, Vermaat JE, van Vierssen W (1987). Seedbank development, germination and early seedling survival of two seagrass species from the Netherlands: *Zostera marina* L. and *Zostera noltii* Hornem. Aquatic Botany.

[60] Coelho H (2011). Fatty acid profiles indicate the habitat of mud snails Hydrobia ulvae within the same estuary: Mudflats vs. seagrass meadows. Estuarine Coastal Shelf. Sciences.

[61] Custódio L (2016). A comparative evaluation of biological activities and bioactive compounds of the seagrasses *Zostera marina* and *Zostera noltei* from southern Portugal. Natural Product Research.

[62] Ramos CS, Parrish CC, Quibuyen TAO, Abrajano TA (2003). Molecular and carbon isotopic variations in lipids in rapidly settling particles during a spring phytoplankton bloom. Organic Geochemistry.

[63] Sanina NM, Svetlana NGoncharova, Eduard Y (2008). Kostetsky. Seasonal changes of fatty acid composition and thermotropic behavior of polar lipids from marine macrophytes. Phytochemistry.

[64] Gordillo FJL, Niell FX, Figueroa FL (2001). Nonphotosynthetic enhancement of growth by high CO2 level in the nitrophilic seaweed *Ulva rigida* C. Agardh (Chlorophyta). Planta.

[65] Sanina, N. M., Goncharova, S. N., Kostetsky, E. Y. Seasonal changes in thermotropic behavior of phospho- and glycolipids from *Laminaria japonica*. In Murata, N. *et al*. (Eds). Advanced Researches of Plant Lipids Kluwer Academic Publishers, Dordrecht, pp. 385–388 (2003).

[66] Allakhverdiev SI, Kinoshita M, Inaba M, Suzuki I, Murata N (2001). Unsaturated fatty acids in membrane lipids protect the photosynthetic machinery against salt-induced damage in *Synechococcus*. Plant Physiology.

[67] Ralph PJ, Durako MJ, Enríquez S, Collier CJ, Doblin MA (2007). Impact of light limitation on seagrasses. Journal of Experimental Marine Biology and Ecology.

[68] Azevedo A, Dias JM, Lillebø AI (2016). Thriving of *Zostera noltei* under intertidal conditions: implications for the modelling of seagrass populations. Marine Biology.

[69] Dias, J. M. *et al*. Influence of mean sea level rise on Ria de Aveiro littoral: adaptation strategies for flooding events and shoreline retreat. In: Green, A. N. and Cooper, J. A. G. (eds). Proceedings 13th International Coastal Symposium (Durban, South Africa), Journal of Coastal Research, Special Issue No. 70, pp. 320–325, ISSN 0749-0208 (2014).

[70] Stefanova A, Krysanova V, Hesse C, Lillebø AI (2014). Climate change impact assessment on water inflow to a coastal lagoon – Ria de Aveiro watershed, Portugal. Hydrological Sciences Journal.

