# Supplementary material for: Effect of spatio-temporal shifts in salinity combined with other environmental variables on the ecological processes provided by Zostera noltei meadows
Source: Sci Rep. 2017 May 2;7:1336. doi: 10.1038/s41598-017-01359-2 (PMC5430980; doi:10.1038/s41598-017-01359-2)
Supplement: Supplementary file 1 — Supplementary information [file 41598_2017_1359_MOESM1_ESM.pdf]

Effect of spatio-temporal shifts in salinity combined with other environmental variables on the ecological processes provided by *Zostera noltei* meadows

Ana I. Sousa[1*], Ricardo Calado[1], Daniel F.R. Cleary[1], Cláudia Nunes[2,3], Manuel A. Coimbra[3], João Serôdio[1] & Ana I. Lillebø[1]

[1] Department of Biology & CESAM – Centre for Environmental and Marine Studies, University of Aveiro, Campus Universitário de Santiago, 3810-193 Aveiro, Portugal

[2] CICECO – University of Aveiro, Campus Universitário de Santiago, 3810-193 Aveiro, Portugal

[3] QOPNA – University of Aveiro, Campus Universitário de Santiago, 3810-193 Aveiro, Portugal

* Corresponding author: anaisousa@ua.pt (A.I.Sousa)

Supplementary Table S1

Zostera noltei trait	Season	Environmental variable	t	*p*
Aboveground biomass	Winter	Temperature	2.663	0.0113*
		pH	-0.698	0.4893
		Salinity	-1.003	0.3221
	Late spring	Temperature	1.129	0.2662
		pH	2.049	0.0477*
		Salinity	0.054	0.9575
Aboveground C pool	Winter	Temperature	2.682	0.0108*
		pH	-0.593	0.557
		Salinity	-1.196	0.239
	Late spring	Temperature	0.744	0.4614
		pH	0.885	0.3818
		Salinity	0.419	0.6774
Aboveground N pool	Winter	Temperature	2.648	0.0117*
		pH	-0.617	0.5406
		Salinity	-0.976	0.3351
	Late spring	Temperature	0.894	0.3769
		pH	1.031	0.3091
		Salinity	-0.203	0.8406
Belowground biomass	Winter	Temperature	2.192	0.0346*
		pH	0.131	0.8965
		Salinity	-0.559	0.579
	Late spring	Temperature	0.751	0.4576
		pH	-0.193	0.8481
		Salinity	1.498	0.1425
Belowground C pool	Winter	Temperature	1.797	0.0803
		pH	0.027	0.979
		Salinity	-0.877	0.386
	Late spring	Temperature	0.603	0.5501
		pH	0.358	0.722
		Salinity	1.624	0.1126
Belowground N pool	Winter	Temperature	1.481	0.1468
		pH	-0.134	0.8944
		Salinity	-0.274	0.7855
	Late spring	Temperature	0.586	0.561
		pH	-0.688	0.4955
		Salinity	0.588	0.5603
PAM - α (α)	Winter	Temperature	1.746	0.0889
		pH	-0.092	0.9268
		Salinity	1.848	0.0724
	Late spring	Temperature	0.499	0.6206
		pH	0.484	0.6311
		Salinity	1.246	0.2204
PAM - ETRmax (rETRm)	Winter	Temperature	0.865	0.3922
		pH	0.429	0.6703
		Salinity	-1.156	0.255
	Late spring	Temperature	0.086	0.9318
		pH	0.018	0.9859
		Salinity	-3.195	0.0028*
PAM – Ek (E_k)	Winter	Temperature	0.269	0.7895
		pH	0.490	0.6267
		Salinity	-1.515	0.138
	Late spring	Temperature	-0.173	0.8635
		pH	-0.233	0.8167
		Salinity	-4.124	0.0002

PAM - Eopt (E_{opt})	Winter	Temperature	0.908	0.3694
		pH	0.662	0.5121
		Salinity	-1.146	0.259
	Late spring	Temperature	-0.968	0.339
		pH	-1.756	0.0874
		Salinity	-1.576	0.1234

Supplementary Table S1 – Statistical results of testing for linear relationships between *Zostera noltei* traits and some environmental variables (sediment temperature, pH and interstitial water salinity), in winter and late spring. Seagrass traits tested are: above- and belowground biomass, above- and belowground C and N pools, and photosynthetic performance (maximum photosynthetic efficiency (α), maximum relative electron transport rate ($rETR_m$), photoacclimation index (E_k) and optimum irradiance (E_{opt}). * refers to a p<0.05 and a significant linear relationship.

Supplementary Table S2
Supplementary Table S2.1.

Zostera noltei aboveground biomass										
	**Site 1**		**Site 2**		**Site 5**		**Site 7**		**Site 10**	
Fatty acid	**Winter**	**Late spring**	**Winter**	**Late spring**	**Winter**	**Late spring**	**Winter**	**Late spring**	**Winter**	**Late spring**
14:0	72 ± 23	56±20	49 ± 15	84±5	46 ± 3	82 ± 60	74 ± 40	83 ± 38	155 ± 112	45 ± 9
16:0	4859 ± 1536	3678±346	3297 ± 422	3584±806	4452 ± 1353	3493 ± 1833	3328 ± 638	3922 ± 960	3405 ± 1146	2710 ± 90
17:0	210 ± 51	205±30	142 ± 15	172±48	178 ± 52	203 ± 115	153 ± 22	243 ± 90	139 ± 18	137 ± 11
18:0	333 ± 49	384±51	266 ± 17	337±67	303 ± 43	337 ± 149	254 ± 38	389 ± 105	275 ± 53	242 ± 3
20:0	165 ± 10	131±31	n.d.	n.d.	n.d.	138 ± 49	169 ± 31	160 ± 32	136 ± 16	105 ± 9
22:0	307 ± 68	207±41	335 ± 37	357±117	240 ± 68	262 ± 60	332 ± 50	251 ± 53	288 ± 92	277 ± 28
24:0	118 ± 50	123±78	115 ± 4	181±34	141 ± 0	105 ± 11	108 ± 26	92 ± 37	119 ± 39	144 ± 21
**∑SFA**	**6063 ± 1549**	**4785±395**	**4204 ± 508**	**4714±1069**	**5313 ± 1314**	**4620 ± 2268**	**4418 ± 712**	**5140 ± 1282**	**4518 ± 1241**	**3660 ± 134**
16:1*n*-7	614 ± 230	405±86	486 ± 98	466±74	543 ± 136	451 ± 296	457 ± 79	534 ± 119	395 ± 138	317 ± 45
18:1*n*-7	139 ± 32	99±9	101 ± 1	116±23	140 ± 58	127 ± 42	104 ± 16	112 ± 22	94 ± 26	91 ± 14
18:1*n*-9	202 ± 79	122±33	258 ± 48	27 2 ±8	172 ± 45	159 ± 100	136 ± 22	250 ± 66	205 ± 54	129 ± 15
24:1*n*-9	212 ± 98	45±23	27 ± 5	54 ±15	19 ± 0	36 ± 30	56 ± 20	30 ± 20	67 ± 21	50 ± 26
**∑MUFA**	**1167 ± 243**	**671±123**	**872 ± 150**	**907 ±86**	**874 ± 238**	**773 ± 456**	**753 ± 127**	**926 ± 209**	**761 ± 235**	**587 ± 90**
18:2*n*-6	3047 ± 1044	2406±478	2450 ± 86	2276 ±1288	3169 ± 1114	2190 ± 381	2368 ± 372	1986 ± 795	1419 ± 654	1468 ± 434
18:3*n*-3	11883 ± 4205	5525±2901	11072 ± 953	5476 ±3037	10867 ± 3186	5014 ± 1746	9441 ± 1478	4713 ± 2117	4204 ± 1791	2735 ± 844
20:4*n*-6	7 ± 0	0 ± 0	11 ± 2	24 ±5	8 ± 0	25 ± 18	10 ± 4	n.d.	19 ± 7	5 ± 2
20:5*n*-3	74 ± 20	31 ± 10	128 ± 29	266 ±36	96 ±31	80 ± 57	116 ± 28	79 ± 55	295 ± 149	52 ± 23
22:6*n*-3	n.d.	14 ± 0	n.d.	22 ±6	28 ± 0	n.d.	n.d.	n.d.	n.d.	1 ± 1
**∑PUFA**	**15011 ± 5264**	**7977 ± 3360**	**13661 ± 931**	**8064 ± 4351**	**14169 ± 4331**	**7309 ± 1895**	**11935 ±1821**	**6777 ± 2825**	**5937 ± 2595**	**4260 ± 1284**
Others	646 ± 163	649 ± 63	549 ± 156	576 ± 35	390 ± 45	496 ± 335	482 ± 126	475±49	551±224	430±70

Supplementary Table S2.2.

Zostera noltei belowground biomass										
	**Site 1**		**Site 2**		**Site 5**		**Site 7**		**Site 10**	
Fatty acid	**Winter**	**Late spring**	**Winter**	**Late spring**	**Winter**	**Late spring**	**Winter**	**Late spring**	**Winter**	**Late spring**
14:0	29 ± 6	31 ± 3	32 ± 11	58 ± 13	49 ± 14	38 ± 24	86 ± 52	24 ± 2	41 ± 11	35 ± 11
16:0	1875 ± 211	2982 ± 1365	2141 ± 225	2550 ± 265	2743 ± 865	2328 ± 1113	3594 ± 2452	2011 ± 497	2066 ± 231	2584 ± 583
17:0	50 ± 6	69 ± 17	53 ± 11	81 ± 5	77 ± 39	62 ± 31	98 ± 61	48 ± 17	60 ± 10	56 ± 13
18:0	172 ± 15	333 ± 163	231 ± 58	358 ± 66	246 ± 57	203 ± 151	254 ± 135	202 ± 26	195 ± 18	204 ± 38
20:0	58 ± 6	101 ± 9	93 ± 6	139 ± 23	73 ± 22	59 ± 21	66 ± 9	81 ± 12	78 ± 9	73 ± 12
22:0	186 ± 16	208 ± 42	196 ± 13	317 ± 40	207 ± 77	161 ± 48	191 ± 18	219 ± 16	188 ± 16	202 ± 36
24:0	173 ± 30	252 ± 84	231 ± 33	378 ± 28	265 ± 105	166 ± 19	149 ± 36	194 ± 46	242 ± 57	210 ± 29
**∑SFA**	**2544 ± 212**	**3975 ± 1437**	**2977 ± 257**	**3881 ± 231**	**3660 ± 1013**	**3017 ± 1351**	**4439 ± 2684**	**2779 ± 467**	**2870 ± 343**	**3364 ± 722**
16:1*n*-7	6 ± 1	12 ± 0	14 ± 9	18 ± 5	18 ± 15	8 ± 1	20 ± 13	6 ± 2	5 ± 2	3 ± 0
18:1*n*-7	336 ± 20	739 ± 501	295 ± 53	371 ± 66	513 ± 191	452 ± 144	476 ± 305	322 ± 39	382 ± 91	432 ± 57
18:1*n*-9	74 ± 9	72 ± 1	65 ± 14	116 ± 21	97 ± 8	88 ± 34	144 ± 82	66 ± 11	118 ± 26	66 ± 14
24:1*n*-9	19 ± 7	32 ± 0	n.d.	36 ± 9	28 ± 15	33 ± 27	42 ± 34	44 ± 34	24 ± 5	22 ± 7
**∑MUFA**	**436 ± 21**	**855 ± 501**	**375 ± 57**	**541 ± 67**	**657 ± 198**	**578 ± 175**	**683 ± 425**	**438 ± 11**	**528 ± 112**	**521 ± 78**
18:2*n*-6	2765 ± 132	3585 ± 722	3300 ± 50	3277 ± 475	4158 ± 1293	2780 ± 1234	4169 ± 2481	2663 ± 307	2982 ± 273	3496 ± 654
18:3*n*-3	1304 ± 16	1274 ± 191	1989 ± 123	1436 ± 196	2207 ± 592	1067 ± 324	2357 ± 1756	1036 ± 133	1601 ± 71	1378 ± 301
20:4*n*-6	0 ± 0	98 ± 84	8 ± 4	n.d.	11 ± 3	n.d.	n.d.	11 ± 0	n.d.	n.d.
20:5*n*-3	9 ± 1	48 ± 39	13 ± 3	17 ± 6	45 ± 9	6 ± 1	18 ± 0	8 ± 1	21 ± 7	22 ± 10
22:6*n*-3	n.d.	n.d.	n.d.	16 ± 12	7 ± 0	n.d.	n.d.	n.d.	n.d.	n.d.
**∑PUFA**	**4078 ± 124**	**5006 ± 1008**	**5310 ± 123**	**4746 ± 659**	**6428 ± 1890**	**3854 ± 1559**	**6538 ± 4225**	**3716 ± 434**	**4604 ± 344**	**4896 ± 944**
Others	293 ± 61	578 ± 350	372 ± 178	503 ± 101	373 ± 163	341 ± 100	537 ± 390	256 ± 77	340 ± 60	353 ± 83

Supplementary Table S2 - Individual esterified fatty acid (FA) concentration (μg g^{-1} dry weight) (mean ± standard deviation, N=3) in *Zostera noltei* biomass in winter and late spring, and at different sites. Table S.2.1 stands for *Z. noltei* aboveground biomass and Table S.2.2 stands for *Z. noltei* belowground biomass. ("Others" refer to the sum of FA 10:0, 11:0, 12:0, 13:0, 14:1, 15:0, 15:1, 16:1*n*-9, 19:3, 20:1, 20:2, 20:3*n*-3, 20:3*n*-6 and 23:0; n.d. stands for not detected).

Supplementary Table S3

Belowground biomass - *Zostera noltei*											
**Site 1 vs site 10**			**Site 2 vs site 10**			**Site 5 vs site 10**			**Site 7 vs site 10**		
**FA**	**Ind. (%)**	**Cum. (%)**	**FA**	**Ind. (%)**	**Cum. (%)**	**FA**	**Ind. (%)**	**Cum. (%)**	**FA**	**Ind. (%)**	**Cum. (%)**
20:4*n*-6	28.65	28.65	24:1*n*-9	18.36	18.36	20:4*n*-6	14.11	14.11	20:5*n*-3	17.63	17.63
20:5*n*-3	13.44	42.09	20:4*n*-6	14.63	33	22:6	11.93	26.04	20:4*n*-6	16.05	33.68
16:1*n*-7	9.02	51.12	16:1*n*-7	12.63	45.63	16:1*n*-7	11.49	37.53	24:1*n*-9	10.26	43.94
24:1*n*-9	5.48	56.59	22:6*n*-3	11.89	57.51	20:5*n*-3	11.22	48.74	16:1*n*-7	9.89	53.82
18:0	5.2	61.79	20:5*n*-3	5.66	63.18	18:0	5.98	54.72	14:0	6.61	60.44
18:1*n*-9	5.17	66.96	18:1*n*-9	5.63	68.81	24:1*n*-9	5.8	60.52	16:0	5.43	65.87
18:1*n*-7	5.14	72.1	14:0	4.58	73.39	14:0	4.72	65.24	17:0	5.15	71.02
20:0	4.84	76.95	24:0	4.47	77.86	18:2*n*-6	4.6	69.84	18:1*n*-7	4.95	75.97
14:0	4.7	81.65	20:0	4.18	82.03	17:0	4.43	74.27	18:3*n*-3	4.77	80.74
24:0	4.47	86.12	18:0	3.82	85.85	16:0	4.4	78.67	24:0	4.31	85.05
17:0	3.97	90.09	17:0	3.19	89.04	18:03	3.9	82.57	18:2*n*-6	4.21	89.27
			22:0	2.88	91.92	18:1*n*-7	3.73	86.3	18:1*n*-9	3.78	93.05
						18:1*n*-9	3.61	89.91			
						20:0	3.45	93.36			

Supplementary Table S3 – SIMPER (similarity percentage) analysis showing the esterified fatty acids (FA) contributing to the differences recorded in the *Z. noltei* belowground biomass from sites 1, 2, 5, 7 and 10 at Mira channel, Ria de Aveiro (Portugal).
